# Meta-omics uncover temporal regulation of pathways across oral microbiome genera during *in vitro* sugar metabolism

**DOI:** 10.1038/ismej.2015.72

**Published:** 2015-05-29

**Authors:** Anna Edlund, Youngik Yang, Shibu Yooseph, Adam P Hall, Don D Nguyen, Pieter C Dorrestein, Karen E Nelson, Xuesong He, Renate Lux, Wenyuan Shi, Jeffrey S McLean

**Affiliations:** 1 Microbial and Environmental Genomics, J. Craig Venter Institute, La Jolla, CA, USA; 2 School of Dentistry, University of California Los Angeles, Los Angeles, CA, USA; 3 Departments of Chemistry and Biochemistry, Pharmacology, and Skaggs School of Pharmacy & Pharmaceutical Sciences, University of California San Diego, La Jolla, CA, USA; 4 Department of Human Genomic Medicine, J. Craig Venter Institute, Rockville, MD, USA; 5 Department of Periodontics, University of Washington, Seattle, WA, USA

## Abstract

Dental caries, one of the most globally widespread infectious diseases, is intimately linked to pH dynamics. In supragingival plaque, after the addition of a carbohydrate source, bacterial metabolism decreases the pH which then subsequently recovers. Molecular mechanisms supporting this important homeostasis are poorly characterized in part due to the fact that there are hundreds of active species in dental plaque. Only a few mechanisms (for example, lactate fermentation, the arginine deiminase system) have been identified and studied in detail. Here, we conducted what is to our knowledge, the first full transcriptome and metabolome analysis of a diverse oral plaque community by using a functionally and taxonomically robust *in vitro* model system greater than 100 species. Differential gene expression analyses from the complete transcriptome of 14 key community members revealed highly varied regulation of both known and previously unassociated pH-neutralizing pathways as a response to the pH drop. Unique expression and metabolite signatures from 400 detected metabolites were found for each stage along the pH curve suggesting it may be possible to define healthy and diseased states of activity. Importantly, for the maintenance of healthy plaque pH, gene transcription activity of known and previously unrecognized pH-neutralizing pathways was associated with the genera *Lactobacillus*, *Veillonella* and *Streptococcus* during the pH recovery phase. Our *in vitro* study provides a baseline for defining healthy and disease-like states and highlights the power of moving beyond single and dual species applications to capture key players and their orchestrated metabolic activities within a complex human oral microbiome model.

## Introduction

The highly diverse oral microbiome represents one of the most studied human microbiomes and is now accepted to have important roles in host health and disease. Oral bacteria are highly impacted by frequent pH drops due to the response of microbial fermentation of dietary carbohydrates and then subsequently returns to normal levels through a combination of bacterial processes and host factors. The particular pH homeostasis that follows a carbohydrate pulse in dental plaque, acknowledged as the ‘Stephan response' as early as the 1940s, has been documented routinely *in vivo*, *ex vivo* and *in vitro* from both dental plaque from healthy human subjects as well as in cultures of single oral isolates (Stephan, 1945; Kleinberg, 1967; Higham and Edgar, 1989; Kleinberg, 2002). In general, for caries-associated plaque samples, pH remains below the ‘critical level for demineralization' for extended periods of time after a carbohydrate pulse, whereas in health-associated plaque, pH may recover more quickly (Marsh and Bradshaw, 1997; Hodgson *et al.*, 2001; Kleinberg, 2002). Frequent or prolonged exposure to low pH may result in carious lesions (enamel demineralization). The realization of the major role of oral bacterial taxonomic and functional diversity in caries led to the development of the 'ecological plaque hypothesis' (Marsh, 1991, 1994; Hodgson *et al.*, 2001). The latter hypothesis proposes that selection of cariogenic bacteria is directly coupled to alterations in the environment that shift the balance of the community (Marsh, 1991). If the pH remains below the ‘critical pH' (value of 5.5) for demineralization for extended time periods (typically after a carbohydrate pulse), a shift in the bacterial populations to more cariogenic organisms that are acidogenic and acid-tolerant (aciduric) can occur (Marsh, 1994; Hodgson *et al.*, 2001). The ecological plaque hypothesis also proposes a homeostasis mechanism, which is responsible for regulating the pH and returning the temporary drop in pH back to neutral (Marsh, 1999). Implicit in these concepts is that disease can be prevented, not only by directly inhibiting acidogenic and aciduric caries-associated pathogens, but also by interfering with the environmental factors driving the selection and enrichment of these bacteria (Bradshaw and Marsh, 1998; Hodgson *et al.*, 2001). A major hurdle to understanding the dynamics of this homeostasis associated with health or disease is the high diversity and the unknown contribution of the very large uncultivated fraction of bacteria in this oral cavity (Lasken and McLean, 2014; McLean, 2014; McLean and Lasken, 2014; He *et al.*, 2015). Although metagenomic approaches enable the sampling of the constituent genomes at a high depth of coverage, these tools cannot be used to infer which bacterial members of the community are active and which pathways are involved in the homeostatic response. With high throughput sequencing of mRNA, expression profiles have been generated representing snapshots of the activity within *in vivo* oral bacterial communities from individual subjects representing healthy and diseased groups (Frias-Lopez and Duran-Pinedo, 2012; Jorth *et al.*, 2014; Benítez-Páez *et al.*, 2014). Owing to the high taxonomic variability and activity of the oral microbiome between study subjects (Benítez-Páez *et al.*, 2014; Human Microbiome Project Consortium, 2012a), it is extremely difficult to track the activity of species at high resolution in a temporal manner. Furthermore, because of the fact that *in vivo* conditions are uncontrollable with respect of nutrient availability, unknown host factors, high inter-person variability and heterogeneous spatial distribution of bacteria, only snap-shots of gene transcription activities are captured. Thus, our current wealth of knowledge on the transcription and metabolism of the oral microbiome is restricted to experiments conducted with single species and difficult-to-interpret *in vivo* samples, and none to our knowledge is directly aimed to investigate this homeostatic process at the transcript and metabolite level.

To address theses major hurdles and to bridge knowledge gaps between *in vivo* and *vitro* studies, a highly diverse oral *in vitro* biofilm model system was previously developed by conducting iterative manipulation of media components and parallel monitoring of the bacterial species diversity (Tian *et al.*, 2010; Edlund *et al.*, 2013). During the development of this model system, the *in vitro* grown biofilms were compared with metagenomes of 242 healthy subjects obtained from natural human samples within the Human Microbiome Project (HMP) (Human Microbiome Project Consortium, 2012a; Edlund *et al.*, 2013). This comparison showed that the model system resembled the oral microbiome at the gene function level (Edlund *et al.*, 2013). The biofilm model proved to be reproducible and stable at both taxonomic and functional levels (Edlund *et al.*, 2013) in contrast to the highly variable species composition of individual subjects and contained ~150 operational taxonomic units taxa, (an operational taxonomic unit is defined as 98% or more in 16S rRNA gene sequence identity), covering 60–80% of the original saliva diversity. Deep sequencing of 16S rRNA genes and metagenomics analyses of the model system provided knowledge of the genetic potential and its community succession. Importantly, when provided a sustained carbohydrate source, the characteristic pH drop followed by a recovery of pH was maintained *in vitro*. This sustained carbohydrate source and subsequent homeostatic recovery is approaching the conditions of dental plaque in anaerobic and anterior regions of the maxilla where food particles may be stuck for longer periods of time and where saliva velocity is low (Van Houte *et al.*, 1991; Arneberg *et al.*, 1997). Using a parallel approach of sampling for mRNA (metatranscriptomics) and global metabolites (metabolomics) temporally along the pH drop and recovery, we acquired a comprehensive dataset generating new insights into the species and activities that influence this fundamental homeostasis process. Here, we report on transcription activities representative of 3376 annotated orthologous groups (KEGG-KOs) of genes that orchestrate an extensive diversity of metabolic pathways, as well as changes in abundance of approximately 400 metabolites between the extra- and intracellular environments. Our replicate libraries covering 18 million to 52 million mRNA reads per sample enabled deep coverage of global community activities and complete transcriptome profiles of 14 key community members that displayed temporal variation in their activity.

## Materials and methods

### Growth media for biofilm establishment and glucose challenge

Chemically defined medium (CDM) was modified after previous protocols (Terleckyj and Shockman, 1975; McLean *et al.*, 2008; McLean *et al.*, 2012). SHI medium was prepared after the protocol of Tian *et al.* (2010). Detailed CDM and SHI media preparation protocols are available (https://depts.washington.edu/jsmlab/downloads/) (Supplementary Methods).

### Sample collection and incubation conditions

Saliva samples were collected and pooled from six healthy subjects, age 25–35 years as described by Edlund *et al.* (2013). The pooled samples were inoculated into SHI medium (Tian *et al.*, 2010) within a sterile 24-well plate. Cell-free saliva was also used for coating wells prior to growing the biofilms (for protocol, see Supplementary Methods). After 16 h of growth in 37 °C in anaerobic conditions, the biofilms were carefully washed twice while in the growth wells with buffered CDM to remove SHI media. Extra careful washing with minimal interruption of biofilms was performed inside the anaerobic chamber. After the washing steps, biofilms were starved (that is, incubated without carbon source) in fresh CDM (pH 7) for 2 h in 37 °C and incubated under anaerobic conditions. After 2 h of starvation, glucose was added at 27.8 mM concentration. Sample collection of biofilms was performed inside the anaerobic chamber at the different pH stages for mRNA and primary metabolite isolation as described below.

### pH monitoring of *in vitro* biofilm growth medium

After glucose amendment, biofilm-growth medium pH was monitored in near real-time within replicate pH-designated incubation wells and measured by combining pH Laboratory Electrodes (EW-05990-65, Cole-Parmer, Court Vernon Hills, IL, USA) with a wireless sensor network platform consisting of a pH transmitter (UWPH-2-NEMA, OMEGA, Stamford, CT, USA) and a pH receiver (UWTC-REC1, OMEGA) (Edlund *et al.*, 2013). pH measurements were monitored and downloaded onto a PC computer by using a TC central software for UWTC (OMEGA). Real-time pH was recorded every 30 s for 12 h within each growth well.

### mRNA isolation, mRNA library preparation and sequencing of *in vitro* biofilms

Samples for mRNA isolation were collected after removing 0.5 ml of the spent CDM and adding 2 volumes of RNAProtect (QIAGEN Inc., Valencia, CA, USA) to each growth well. Sequencing was carried out at the (J. Craig Venter Institute) JCVI Joint Technology Center (JTC) by using an Illumina HiSeq 2000 platform (San Diego, CA, USA) (100 bp paired end reads). mRNA sample concentrations were normalized at JTC prior to sequencing. Detailed protocols and references are provided in Supplementary Methods. Quality filtered mRNA read libraries have been submitted to NCBI under BioProject ID PRJNA264728.

### mRNA read mapping and identification of active species in biofilms

To generate a reference genome data set representative of the highly diverse oral microbiome, assembled contigs representing approximately 5000 bacterial species were downloaded from the HMP database (Chen *et al.*, 2010; Human Microbiome Project Consortium, 2012b) and the JCVI in-house curated database (phyloDB). In total, 2 90 091 contigs were downloaded (10 182 from HMP, 8953 from HOMD, 2 70 956 from phyloDB). Contigs shorter than 500 bp and that showed a >60% base pair skew (that is, a particular nucleotide was represented in a contig at a frequency of 60% or higher) were disregarded. mRNA sequencing libraries, representing the different pH stages were individually mapped to this reference data set by using the Burrow-Wheeler Aligner program (Li and Durbin, 2009). No paired reads restriction was enforced and the default BWA mapping option was applied, resulting in the identification of mRNA reads with ⩾96% sequence identity to the reference-genome data set. Bacterial reference genomes that provided the highest coverage of mRNA reads and also represented different genera were selected for individual genome analysis to enable studies of gene transcription at a species level.

mRNA sequencing libraries representative of each pH stage were mapped to each of the following genomes; *Streptococcus salivarius* CCHSS3, *S. vestibularis* F0396, *Streptococcus* sp. C-150, *S. mitis* bv_2_str F0392, *S. thermophilus* LMD-9, *S. parasanguinis* ATCC15912, *S. sanguinis* ATCC 49296, *S. agalactiae* ATCC 13813, *Veillonella atypica* ACS-134-V-Col7a, *V. dispar* ATCC 17748, *Lactobacillus fermentum* IFO3956, *Klebsiella* sp. MS 92-3, *Gemella haemolysans* ATCC 10379 and *Fusobacterium* sp. 2_1_31. Paired reads restriction was enforced meaning that both forward and reverse sequence read was required to map at the same genome location to generate a positive BWA mapping result. The default BWA mapping option was applied and matches were only reported for reads with two mismatches or less in the first 32 bases at the beginning of the reads. The ‘–n' parameter was set to 0.04 allowing BWA to only report on reads with ⩾96% identity over 90% read length. Detailed processing of the mRNA-read mapping analyses is available in Supplementary Information.

### Global transcriptomic profiles

To gain a deeper understanding at a community level, of gene expression responses associated with the Stephan-curve, we applied the non-redundant open reading frame (ORF)-reference dataset to classify and quantify mRNA reads from all nine mRNA libraries at different levels of functions. Differential gene expression analysis at a community level was visualized using a network approach that compared the fold change of DESeq read count data between the pH stages (pH 4.2 vs neutral pH 7 and pH 5.2 vs 4.2) at Kyoto Encyclopedia of Genes an Genomes (Kanehisa *et al.*, 2008) (KEGG) Orthogroups (KO)-group level (Supplementary Dataset S1). This analysis showed that like the primary metabolites, the global community response of transcription activity changed dramatically (Supplementary Figure S2). In total, 3314 KO groups had mRNA reads that aligned (Supplementary Dataset S1) and 420 of these KO groups showed a significant increase following false discovery rate correction (Materials and methods) as pH dropped from neutral to 4.2 (Supplementary Dataset S1). For detailed protocol and references, see Supplementary Information. Changes in bacterial growth across pH stages were analyzed based on normalized expression of cell division genes as described in Supplementary Methods. Gene expression activities for the specific alkali generating pathways were also analyzed across pH stages as described in Supplementary Methods.

### Primary metabolite analyses with gas chromatography–time-of-flight mass spectrometry

All mass spectrometry analyses of primary metabolites were conducted at the NIH West Coast Metabolomics Center, UC Davis Metabolomics Core Laboratory, the Fiehn Laboratory, Davis, CA, USA. A detailed description of sample preparation and the detailed gas chromatography-mass spectrometry protocol is available in Supplementary Information.

### Global small molecule network analyses with liquid chromatography mass spectrometry

Briefly, six replicate *in vitro* biofilm samples were collected from each pH stage 7, 4.2 and 5.2 and were analyzed from global small molecules. Replicate MS data files in mzXML format (three replicates per pH stage) were uploaded onto the UCSD hosted Global Natural Products Social Molecular Networking webserver (http://massive.ucsd.edu/ProteoSAFe/static/massive.jsp?redirect=auth). For detailed protocol and references, see Supplementary Material.

## Results and discussion

### Global metabolite profiles define unique metabolic states

**Figure 1:**
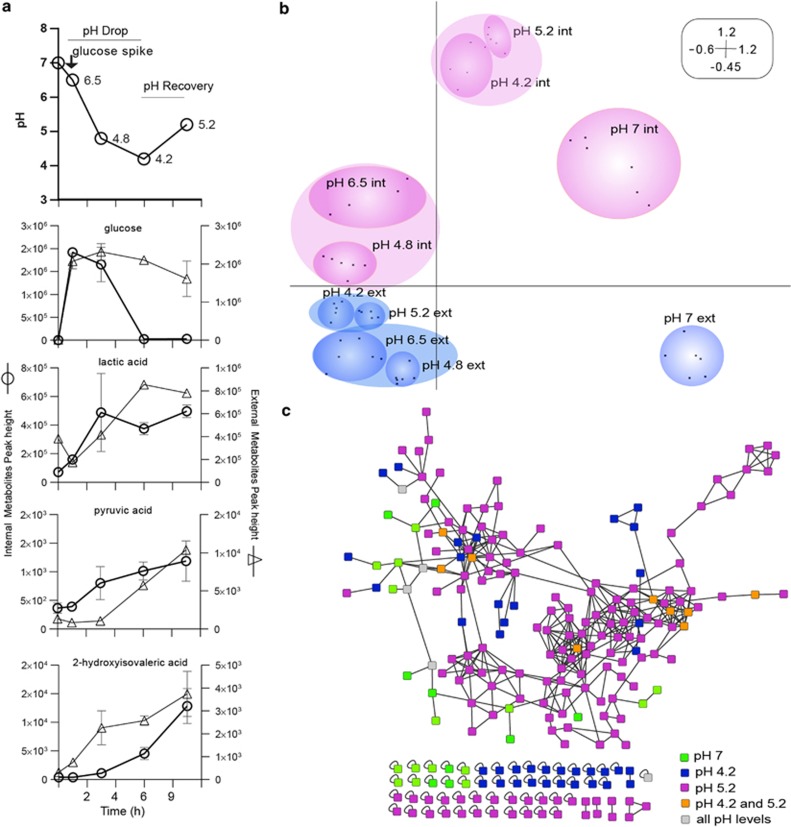
Primary metabolite and small molecule fingerprints are discernable temporally and cluster by environment. (**a**) Dynamic changes of extracellular pH and 133 metabolites with annotations in reference libraries. Metabolite measurements derive from both extracellular (open triangles) and intracelular (open circles) fractions before and after a glucose pulse. (**b**) Similarities between metabolite profiles obtained by gas chromatography-mass spectrometry (GCMS) from the extracellular (ext) and intracellular (int) biofilm environments at the different pH stages were analyzed with correspondence analyses (CA). The first ordination axis explains 53% of the variation of the data set and the second ordination axis explains 32% of the variation. Six replicate samples (depicted as small black squares in ordination diagram) from GCMS analyses were included to represent each environment and pH stage. (**c**) Tandem mass spectrometry (MS/MS) network of secreted mall molecules from biofilms at pH stages 7, 4.2 and 5.2. Molecular networks were obtained by spectral alignment as described in Watrous *et al.* (2012). Molecules with no structural homologues were included as singletons in the lower section of the network. The network comparison is based upon the similarity cosine scoring of MS/MS spectra and the visualization of those relationships. A single chemical species is represented as a colored node and the relatedness between spectra are represented as edges.

**Figure 2:**
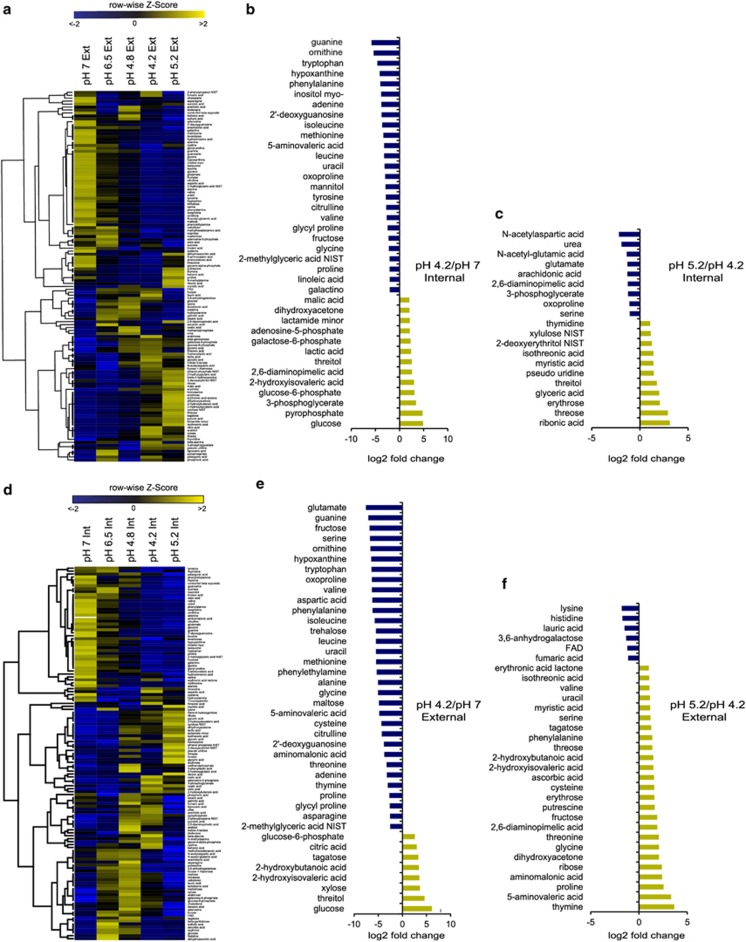
Global metabolite profiles reflect temporal changes within extra- and intracellular biofilm fractions. (**a** and **d**) Hierarchical cluster analyses of metabolites obtained from (**a**) intracellular extracts of biofilms across different pH stages and (**d**) supernatant (extracellular) extracts of biofilms across different pH stages by gas chromatography-mass spectrometry. Peak height data for each identified compound within each row were normalized using the *z*-score. Yellow indicates high relative metabolite concentrations (*z*-score values ⩾2); blue indicate low relative metabolite concentrations (*z*-score values ⩽2); black indicate the median *z*-score values. (**a** and **d**) Metabolites showing significant fold change comparisons between pH stages (Fold change <log_2_ or >log_2_). (**b**) Relative metabolite differences of the intracellular environment between the lowest pH stage (pH 4.2) and neutral pH (prior to glucose amendment). (**c**) Relative supernatant differences between the lowest pH stage and the recovery stage (pH 5.2) representing intracellular metabolites changing during the pH recovery phase. (**e**) Relative differences in metabolites from the extracellular environment between the lowest pH stage (pH 4.2) and neutral pH (prior to glucose amendment). (**f**) Relative differences in metabolites from the extracellular environment between the lowest pH stage and the recovery stage (pH 5.2).

Phenotypic, transcriptional and metabolic responses of the *in vitro* biofilm community were determined for time points representing various pH stages during glucose catabolism (Figure 1a). Importantly, although the rate and degree of pH change may differ depending on many factors, including the sugar concentration, intraoral site and saliva host buffering (Kanapka and Kleinberg, 1983), the trend in pH representing a general homeostatic mechanism is consistent and an important feature of dental plaque. This feature is seen both using *in vivo* measurements and *in vitro* experiments. It is important to highlight that the rate of change in pH, within the *in vitro* biofilm model we present here, may be more representative of dental plaque in anaerobic and anterior regions of the maxilla where food particles (carbohydrate source) may be stuck for longer periods of time and where saliva velocity is low (Van Houte *et al.*, 1991; Arneberg *et al.*, 1997). Our sampling strategy was designed to capture gene expression and metabolites during periods after major shifts in metabolism, which includes the shift from no sugar to after sugar addition when the pH is at a minimum and the shift from low pH to pH 5.2, capturing the changes during the recovery phase. Samples representing both the extracellular metabolites (M_ext_) and the intracellular metabolites (M_int_) (that is, supernatants without cells and lysed cells without supernatant, respectively) of biofilms were analyzed from several pH stages along the Stephan curve before and after glucose amendment. After glucose was added to the biofilms, significant shifts in the relative abundance of numerous metabolites occurred across the full pH spectra (Figure 2). Of the total 402 masses detected in gas chromatography-mass spectrometry, 133 had matching annotations (Figure 1b) when compared with the 1083 library compounds at the NIH-West Coast Metabolomics Center (Supplementary Table S1). Glucose decreased drastically in M_int_ and was low at pH 4.2, whereas it remained high in M_ext_ at the later pH stages. These results show that glucose metabolism was inhibited presumably by low pH and that glucose import was also regulated (Figure 1a). Characteristic of oral plaque with access to carbohydrates, lactate concentrations increased both in M_ext_ and M_int_ across the pH stages; however, a dip in production was observed at pH 4.2 indicating that a metabolism shift occurred and that heterolactic fermentation was becoming more abundant resulting in the production of other organic acids. Two such examples from the large number of temporally varying compounds (Figure 2) include pyruvic acid and 2-hydroxy isovaleric acid that are products of homolactic and heterolactic fermentation (Figure 1a). Multivariate statistics analyses showed that metabolite profiles representing all pH stages for the M_ext_ and M_int_ clustered separately showing highly different metabolic activities between the inside and outside of the biofilm cells (Figure 1b). All six replicates clustered together for each pH stage (Figure 1b) showing the reproducibility of metabolite profiles mirrors the reproducibility in the species carriage and abundance previously shown in this model (Edlund *et al.*, 2013). The analysis demonstrates that the metabolite profiles representing the neutral pH stages were unique and that at pH 6.5 and 4.8 (1 h and 3 h after glucose amendment, respectively), the metabolite profiles were highly similar for their respective environment (*r*-values=0.98 and 0.99, respectively) (Figure 1b). The later pH stages (pH 4.2 and 5.2) also clustered together for each environment showing that their metabolite diversity is similar (*r*-values=0.97 for clusters representing both environments) (Figure 1b). However, a more detailed and in-depth comparison of the fold change differences of individual annotated metabolites across all pH stages revealed that several metabolites increased and decreased in relative abundance as pH recovered (Figure 2).

Secreted small molecules within the size range of 50–1600 Dalton were also extracted from biofilms and subjected to tandem mass spectrometry to target pH-specific molecular masses that represent peptides, fatty acids and glycolipids, which may correspond to important signaling and/or antagonist molecules within the community. The GNPS molecular network analyses approach was applied (Supplementary Methods) to compare and score mass spectrometry fragmentation patterns of molecular masses in all samples and allowed for the observation of unique molecules produced at each pH stage (Figure 1c). Taken together, the analyses of metabolic profiles (Figure 1b) and molecular network analyses of tandem mass spectrometry spectra (Figure 1c) support that differences exist in the metabolic output between neutral pH 7 and recovery pH 5.2, and low pH states (pH 4.2), which is information that may ultimately be used to inform and interpret similar data from future clinical studies of caries disease (Figure 1b).

Global temporal analyses of the observed metabolites revealed clear changes both in metabolite identity and their relative presence between the stages of pH 7 and pH 4.2 (pH drop) and pH 4.2 to pH 5.2 (recovery phase) (Figure 2). At neutral pH, after 2 h of starvation in minimal medium, the biofilms harbored several free amino acids both M_ext_ and M_int_, which reflect the potential of amino acids to serve as important carbon and nitrogen sources during carbohydrate-limited conditions (Figures 2b, c, e and f). The majority of these were significantly depleted after glucose was added (Figure 2). At pH 6.5, 1 h after glucose amendment, a drastic change in the relative abundance of metabolites was clear and at the lowest pH stage (4.2), for both M_ext_ and M_int_, organic acids such as lactate, 2-hydroxyisovaleric acid, 2-hydroxy butanoic acid, glyceric acid and pyruvic acid increased significantly, reflecting activity of bacterial community members with aciduric and acidogenic metabolic capacities (Figure 2). As pH recovered to 5.2 in the extracellular environment the triose dihydroxy acetone (also known as glycerone) and organic acids (for example, 2-hydroxyisovaleric acid, erythronic acid lactone, isothreonic acid, 2-hydroxy butanoic acid) continued to increase. Some of these metabolites have higher pKa values than lactate, which is the most abundant acid at the lowest pH stages, and therefore they are highly likely to contribute to the pH recovery. Dihydroxy acetone increased 10 times at pH 5.2 and has a pKa value of 13.49, which suggests that alkaline metabolites indeed are present and have a potential to impact the low pKa of lactate and 2-hydroxy butanoic acids (Figure 2f). Also, free amino acids (proline, glycine, cysteine, valine and serine) with higher pKa values than lactate increased significantly in the extracellular environment at pH 5.2. These results are in line with previous findings where alkaline organic acids, such as acetate were identified in mono-species growth cultures and in a mixed culture of nine species as the pH increased (Bradshaw *et al.*, 1989). In addition to these findings, metabolites that belong to known pH-tolerance and alkali-generating pathways (arginine, ornithine, citrulline, glutamate, serine, threonine and urea) varied dramatically. The genes encoding these particular pathways showed significant differential transcription activities in response to the changing pH and are investigated in more detail in the following sections. The primary metabolites that we detected here, dihydroxy acetone and 2-hydroxyisovaleric acid, were for the first time (together with more alkaline amino acids) directly associated with pH-neutralizing changes within the oral community and should be further investigated as to their impact on plaque pH. The role of dihydroxy acetone is of specific interest as its pKa value is 13.49. To ultimately determine the ecological roles of these identified molecules, which also represent potentially new oral microbiome-modulating compounds, future studies need to focus on compound isolation, structure elucidation and functionality testing both in the natural ecosystem and in a clinical setting.

### Community-level expression profiling results

With the goal to elucidate functional differences between pH stages at community level, we mapped paired-end reads, generated by sequencing the corresponding mRNA libraries, to a non-redundant ORF dataset. This ORF data set consisted of 2 288 459 unique KEGG-annotated ORFs representing genes extracted from sequenced bacterial genomes, *de novo* assembled genes from our previously described metagenomes from our *in vitro* grown biofilms (Edlund *et al.*, 2013) as well as *de novo* assembled cDNA reads from this study. Mapping of individual replicate mRNA libraries to the ORF dataset resulted in a high mapping success (68–82% of the reads mapped) (Supplementary Table S2). Mapping counts for each ORF was normalized with the DESeq model that takes into account both technical and biological variability and is not biased towards gene length, GC content and dinucleotide frequencies which other known RNA-Seq normalization models can be (Anders and Huber, 2010). Notably, high correlation values were observed between replicates (Table 1,Supplementary Figure S1) with the starvation phase prior to glucose at pH 7 (*r*=0.99) and the pH recovery end point pH 5.2 (*r*=0.95–0.99). The lowest pH stage exhibited slightly higher variability between replicates (*r*=0.81). Overall, this strong correlation in transcript counts, as well as the clustering of metabolites between replicate samples, is likely a direct result of the stability in species and metabolic function observed previously between replicate wells of this same biofilm community (Edlund *et al.*, 2013). To further verify how representative our metatranscriptome libraries were with respect to mRNA-read mapping success across coding genes representing completely sequenced genomes, we calculated the total numbers of coding DNA sequences that had aligning mRNA reads for each reference genome and for each pH stage (Table 2). The coverage was high for all genomes that were included in this study as 84–100% of the total number of coding DNA sequences had matching mRNA reads at the different pH stages (Table 2). On the basis of these results, we show that overall gene expression activity in this study was well represented by the reference genomes and did not only reflect certain groups or families of genes (Table 2). The global transcriptomic profile and how these activities changed across pH stages at a community level were generated and analyzed at a broad functional level (Supplementary Figure S2). In total, 3314 KO groups had mRNA reads that aligned (Supplementary Table S1) and 420 of these KO groups showed a significant increase following false discovery rate correction (Materials and methods) as pH dropped from neutral to 4.2 (Supplementary Figure S2,Supplementary Dataset S1). A detailed discussion of the pathway changes seen is presented in Supplementary Information.

### Temporal gene transcription activities across species and genera

**Figure 3:**
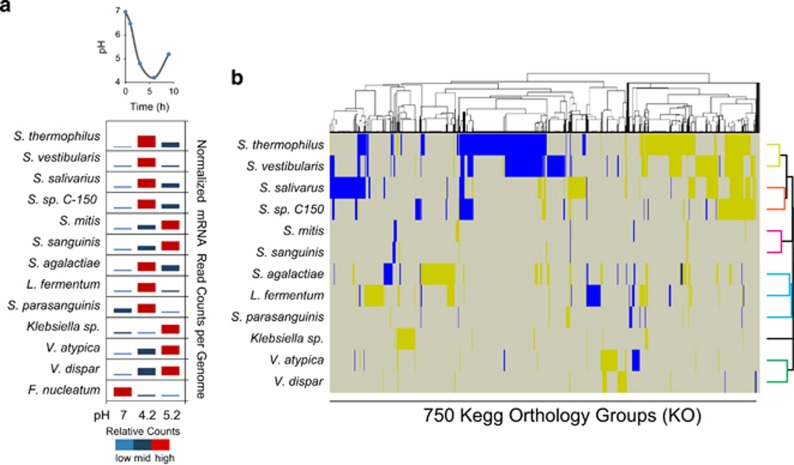
Key species responses at mRNA read level and KEGG-Orthology (KO) level in reaction to glucose amendment followed by a drastic pH drop. (**a**) Normalized mRNA read counts that mapped to reference genomes at the different pH stages. (**b**) mRNA reads that mapped to coding DNA sequences of reference genomes could be classified by using the KO system to a high extent (Supplementary Dataset S1). Differential expression was calculated between pH 4.2 and 7 for each KO group and genome. Hierarchical cluster analyses grouped reference genomes together according to similarities in up- (yellow) and down-expression (blue) of KO groups. Shared KO groups across this set of genomes that showed no changes in expression are indicated in grey.

Reads from each replicate mRNA library were mapped to 14 reference genomes which were chosen based on significant differential expression of their genes (following false discovery rate correction) across pH stages and which were also representative of distinct oral genera (that is, *Streptococcus, Veillonella, Lactobacillus*, *Klebsiella*, *Gemella* and *Fusobacterium*) (Table 2). The overall results show both functional differences and overlaps between phylogenetically distant community members in response to acidification and glucose amendment (Figure 3). Also, species within the *Streptococcus* genus behave very differently and are active at different pH stages showing the vast functional variability within this highly diverse genus, which is important to consider in future attempts to develop treatment strategies for caries disease. *S. thermophilus*, *S. vestibularis, S. salivarius* and *Streptococcus* sp. C-150 showed more similar KO functions and had the highest activity at pH 4.2. Similarities in responses indicate mutualistic and/or synergistic interactions between these four species in low pH conditions, which needs to be addressed in future research. Functional clustering at the KO level was also observed for *S. mitis* and *S. sangunis* at the recovery pH 5.2, which indicate species-specific interactions that may have important roles for the recovery phase (Figure 3b). *S. parasanguinis* and *S. agalactiae* seem to be the most different *Streptococcus* species as their functional responses clustered most closely to *L. fermentum*. These three species also show the highest activity at pH 4.2 (Figure 3b). The *Klebsiella* sp. does not match any other genome at KO level. Members belonging to the *Klebsiella* genus are highly understudied in the context of the interactions that occur within the complex oral microbiome. A previous study shows that *K. pneumonia* is present in a small proportion of healthy adult subjects within oral cavity including saliva and supragingival plaque at a low abundance (0.01–1%) depending on the oral site (Conlan *et al.*, 2012). The gene expression response we observed for the *Klebsiella* sp. in the *in vitro* biofilm community provides us with a unique opportunity to acquire a deeper knowledge of this rare and possibly clinically important community member. The two *Veillonella s*pecies are highly similar at the KO group level, however, functional differences were observed at gene level, which will be discussed later. *Fusobacterium* sp. was highly active at pH 7 and showed a clear decrease in activity at the lower pH stages. The numbers of significant differentially expressed KO groups were too few for this species at the lower pH stages and therefore it is not included in the cluster analyses in Figure 3b. Detailed transcriptome response at the gene level for each species is discussed in the context of key pathways. All individual transcriptome datasets on the differential expressed genes and functional levels are available in Supplementary Dataset S2 with additional discussion of gene expression results for key species in Supplementary Information. To address whether the observed gene transcription patterns were influenced by growth over the experimental time period within the bacteria we chose to study in detail, we additionally selectively analyzed transcriptional changes of cell-division genes (Supplementary Dataset S2). The included reference genomes for these calculations were those that showed either high activity or changed significantly in fold change across pH stages. No significant fold changes could be observed for the transcribed cell-division genes, suggesting that growth was not a significant factor for determining the set of significantly differential expressed genes across the pH stages (Supplementary Dataset S2).

### Highly diverse activities of alkali-generating pathways within oral bacteria

**Figure 4:**
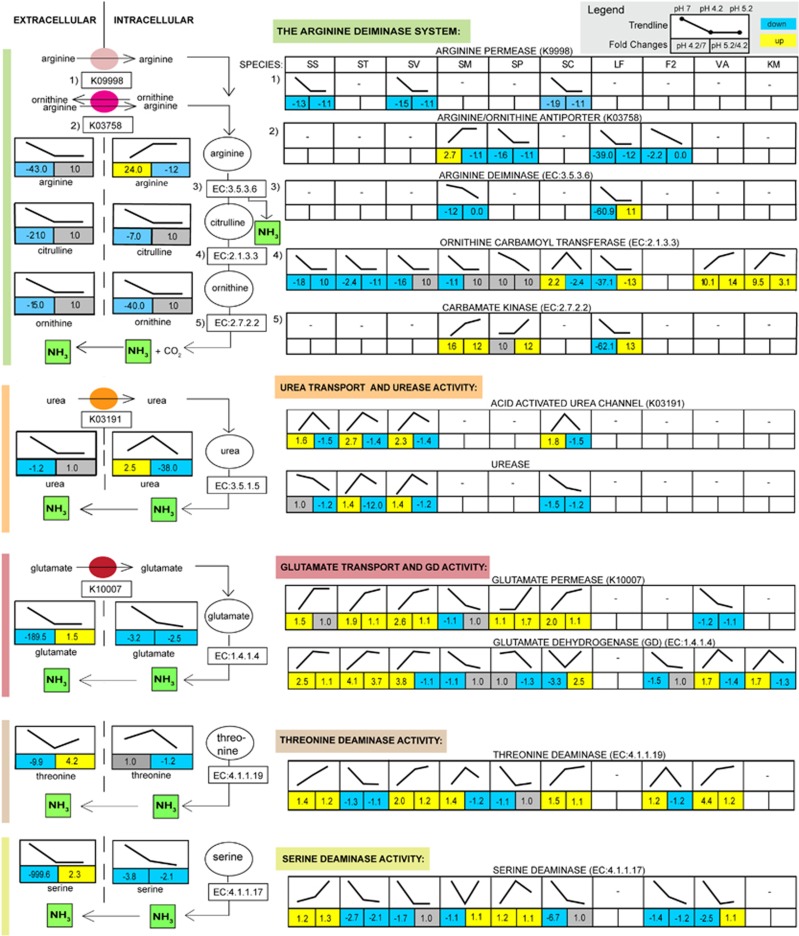
Gene transcription activity and metabolite fluxes associated with alkali-generating pathways at different pH stages within the *in vitro* biofilm. Relative metabolite abundance was determined by using gas chromatography-mass spectrometry of intracellular biofilm extracts and growth media of biofilms (extracellular growth extracts). Differential transcription activity of key enzymes at the different pH stages in alkali-generating pathways, representative of different community members was determined by mapping of mRNA reads to reference genomes. Reference genomes correspond to the species that recruited the most mRNA reads and represent; *S. salivarius* (SS), *S. thermophilus* (ST), *S. vestibularis* (SV), *S. mitis* (SM), *S. parasanguinis* (SP), *Streptococcus* sp. C-150 (SC), *L. fermentum* (LF), *Fusobacterium* sp., *V. atypica* (VA) and *Klebsiella* sp (KM). Significant transcription activities were identified for the ADS, the urease enzyme, the glutamate dehydrogenase enzyme and the threonine and serine deaminase enzymes. Transcription activity of associated metabolite transporters was also determined. −, no transcription activity was detected.

Caries is known as a multi-factorial disease and low pH is its primary determinant. Consequently, a major focus of caries research has been on characterizing acid-generating bacteria and the mechanism of acid resistance of cariogenic bacteria and less attention has been given to alkali-generating processes that are associated with enamel remineralization and health. Thus far, transcription activity of these pathways in dental plaque is restricted to only a few pathways within single strains (Margolis *et al.*, 1988; Van Wuyckhuyse *et al.*, 1995; Burne and Marquis, 2000; Clancy *et al.*, 2000) therefore, it is of great interest to study these processes in a whole-community context. Alkali production in the form of ammonia can inhibit tooth demineralization by neutralizing glycolytic acids and it is hypothesized that this promotes healthy oral plaque by favoring the persistence of an alkalinogenic microflora (Nascimento *et al.*, 2009). Alkalinization *via* ammonia production in particular is a well-studied mechanism to increase the cytoplasmic pH and the environment. This protects the cells from acid killing, allows ATP generation and maintains a balanced ΔpH for enhanced bioenergetics and is thought to have a major role to favor groups of what are thought of as health associated bacteria. It has been proposed that increased caries risk is associated with reduced alkali-generating capacity of the bacteria colonizing the oral cavity (Nascimento *et al.*, 2009). In this study, we identified and report on a number of alkali-generating processes and their associated metabolic precursors and byproducts, which had highly varied transcriptional activity across pH and across species (Figure 4).

The highest transcription activities across all pH stages represented genes encoding glutamate dehydrogenase (GD), urease, and threonine and serine deaminase enzymes (Figure 4). The metabolic process where urea is converted to ammonia and carbon dioxide by a nickel-dependent urease (EC:3.5.1.5) was highly active at all pH stages but showed a varying response across four *Streptococcus* species (*S. salivarius, S. vestibularis, S. thermophilus, Streptococus* sp. C-150) suggesting a differential regulation of this enzyme (Figure 4). The common response for all four species was that the urease activity decreases as pH recovered to 5.2 (Figure 4). A high affinity nickel transporter protein (K07241) was also observed to be highly active at the lowest pH demonstrating the importance of urease activity in neutralizing pH in our system (Supplementary Dataset S1). The four *Streptococcus* species also harbored the acid-activated urea channel protein (K03191) that imports urea into the cells and that was highly active in pH 4.2. Its activity decreased as pH recovered to 5.2 (Figure 4) showing an important role of this channel protein and the urease activity in generating a more alkaline environment. In line with these observations, measurements of urea concentrations show that as the pH decreased, urea increased internally indicating the import of urea into the cells likely *via* the mechanisms described above. Urea concentrations peaked internally at pH 4.2 and utilization was evident from pH 4.2 to pH 5.2 (Figure 4) although not statistically significant because of variability in the concentrations of this particular metabolite. *S. parasanguinis* and *S. mitis* do not harbor urease encoding genes, however, these two species used an alternative pH-neutralizing strategy, namely glutamate reduction *via* the GD enzyme (EC:1.4.1.4) to form ammonia at all pH stages (Figure 4). In fact, this pathway was used by all analyzed species, except *L. fermentum* that did not harbor the GD encoding gene. GD activity increased as pH decreased to 4.2 for *S. salivarius, S. thermophilus, S. vestibularis, V. atypica* and *Klebsiella* sp., whereas its activity decreased for *S. mitis, Streptococcus* sp. C-150 and *Fusobacterium* sp. (Figure 4). For *S. salivarius, S. thermophilus* and *Streptococcus* sp. C-150, GD activity increased as pH recovered to 5.2, whereas it decreased or remained at the same level for *S. vestibularis*, *S. mitis*, *S. parasanguinis*, *V. atypica, Fusobacterium* sp. and *Klebsiella* sp. The rapidly decreasing metabolite concentrations of glutamate internally (*P*: 0.007; *n*=6) and externally (*P*: 0.0001; *n*=6) (3-fold change and 180-fold change from pH 7 to pH 4.2, respectively) indicate the utilization of this amino acid (Figure 2,Supplementary Table S1).

All *Streptococci* genome representatives as well as the *V. atypica* and *Fusobacterium* sp. genome representatives used threonine deamination which results in the formation of ammonia *via* the enzyme threonine deaminase (EC:4.3.1.17), revealing another potentially important alkali-generating process (Figure 4). The threonine deaminase activity was relatively stable across the different pH stages but increased slightly for *S. salivarius*, *S. vestibularis, Streptococcus* sp. C-150 and *V. atypica* as pH dropped to 4.2 and recovered to 5.2. Threonine concentration profiles indicate external concentrations rapidly change between pH 7 and pH 4.2 (10-fold) however, internally the concentrations are varied but indicate depletion initially (Figure 2f) and possible utilization during the recovery phase. The metabolite data also indicate utilization of available serine pools during the pH decrease externally and internally (~1000-fold and ~4-fold, respectively) (Figure 2c). The serine deaminase (EC:4.3.1.17), which is involved in the deamination of serine can contribute to the observed increase in pyruvate pools (Figure 1,Figure 2) and generates ammonia as a byproduct. The enzyme was constitutively expressed in *S. salivarius, S. mitis* and *S. parasanguinis* across all pH stages. However, for *Streptococcus* sp. C-150, *S. thermophilus, S. vestibularis, Fusobacterium* and *V. atypica,* it decreased as pH dropped to 4.2. The serine deaminase was absent in *L. fermentum* and *Klebsiella* sp. Other important ammonia-generating processes that were highly upregulated in the community were membrane proteins involved in ammonia gas conduction (members of the Amt family, K03320), which were highly upregulated at pH 4.2 and 5.2 in *S. salivarius* and S*. vestibularis* (Supplementary Dataset S2). These enzymes have a central role in excretion of ammonia to the extracellular environment (Andrade and Einsle, 2007) and were likely to contribute to the pH recovery we observed here.

The most well-studied alkalinization pathway is the arginine deiminase system (ADS) pathway, which we examined in detail at the metabolite and at the transcriptional level for multiple species here (Figure 4). For many of the studied species, the expression of the enzymes in this pathway was unexpectedly downregulated at low pH. A reason for this could be that this may be a mechanism necessary to avoid over-alkalization of the internal environment. Also, the responses of these pathways were highly dynamic and their activities seem to be regulated in a highly species-specific manner of which very little is known thus far. Our research reveals that in addition to the well-studied ADS pathway, other alkali-generating pathways are active in common abundant oral species. Overall, based on the observed relative abundance measures of each gene encoding for the enzymes in these pathways (Supplementary Dataset S1) and changes in the corresponding gene transcriptional activity across pH stages (Figure 4), we propose that the ADS pathway is important in the early stages for bacteria such as *S. mitis* and *L. fermentum* (those lacking urease genes) to survive acid stress. In a previous study of a 10 species *in vitro* model of a constant-depth film fermenter, it was shown that urease activity was likely the most influential process in preventing the emergence of a cariogenic microflora (Shu *et al.*, 2003). These authors proposed that the loss of sufficient quantities of urease resulted in acidification of the biofilms, leading to loss of community diversity and the emergence of an aciduric flora. In line with these findings, it is likely that the urease activity in our *in vitro* model was also reflecting a health-associated response as the pH recovered after the major drop to 4.2 (Supplementary Dataset S1). Whereas the activities of glutamate dehydrogenase as well as the serine deaminase may become important for many oral bacteria during the recovery phase given the continued increase in expression of these enzymes and their abundance (that is, there are multiple copies of serine deaminase in most of the genomes we studied) (Supplementary Dataset S1). However, before we can fully predict which enzyme has the most critical role in these processes in the *in vitro* model and ultimately *in vivo*, each enzyme's substrate specificity and reaction rate have to be empirically determined for each bacterium and condition. A detailed discussion of ADS activities in the biofilm community is provided as Supplementary Information. Further experiments to continue to dissect these pathways and their contribution are underway.

### Newly discovered contributions of *Veillonella* at low pH and during pH recovery

The functional role of *Veillonella* in alkali-generating processes has not been previously reported, to our knowledge. Earlier studies suggest that the two more known species, *V. dispar* and *V. atypica*, are functionally highly similar (Sato *et al.*, 1997; Igarashi *et al.*, 2009). In contrast, we show that *V.dispar* and *V. atypica* respond differently to acid stress by using entirely different enzymes that have important roles in pH recovery. At the lower pH stages, *V. dispar* shows significant upregulation (3.7-fold) of a gene encoding an acetyl-CoA hydrolase (EC:3.1.2.1) and a meso-diaminopimelate D-hydrogenase (EC:1.4.1.16) (fivefold), which are key enzymes in ammonia generating pathways (Supplementary Dataset S2). *V. atypica* shows upregulateion of genes encoding the ADS, a glutamate dehydrogenase and serine and threonine deaminases which are absent genes in the *V. dispar* genome (Figure 4). Also, by analyzing gene expression of the glycerol dehydrogenase (EC:1.1.1.6) enzyme that catalyzes the formation of the highly alkaline hydroxy acetone metabolite, which increased across pH stages, we identified that this enzyme was highly active as pH decreased (two- to threefold increase across pH stages) in *V. atypica* and *V. dispar* (Supplementary Dataset S2). On the basis of these results, we propose that both *Veillonella* species likely contribute to the production of this highly alkaline metabolite at the later pH stages. In *V. atypica,* a proton transporting ATPase (K02108) that acts on acid anhydrides and transports protons out of cells (KEGG pathway EC: 3.6.3.14) and a hydrogenase (K06281, K06282; small and large subunit, respectively) that generates hydrogen gas (KEGG pathway EC:1.12.99.6) were also significantly upregulated (2.4- and 2.5-fold, respectively) at the lowest pH stage indicating an important mechanism for maintaining intracellular pH levels in an acid-stressed environment (Supplementary Dataset S2). On the basis of these results, we propose that *V. dispar* and *V. atypica* metabolic activities were positively stimulated by the presence of lactic acid and low pH conditions induced by glucose-spiking. This is the first report to our knowledge, which confirms and characterizes the gene activity of *Veillonella* at low pH. Other important functions that are not related to alkaline-generating pathways or directly involved in acid metabolism and that changed significantly across the pH stages for the *Veillonella* genus are discussed in Supplementary Information.

### Multiple low pH tolerance mechanisms are active in *Lactobacillus*

Members of the *Lactobacillus* genus are known participants in the development of dental caries (Alaluusua *et al.*, 1987; Teanpaisan *et al.*, 2007; Gross *et al.*, 2010). They are microaerophilic, Gram-positive organisms that ferment hexose sugars to produce primarily lactate. *Lactobacilli* inherently acidify their environment by producing lactate very rapidly, self-imposing acid stress. The need to respond to acidification has resulted in a multitude of adaptations that are competitive advantageous for this group of organisms. The use of proton translocation F-ATPases (Bender *et al.*, 1986; Bender and Marquis, 1987), the ADS (Burne *et al.*, 1989), malolactic acid fermentation (Pilone and Kunkee, 1970) and increased biosynthesis of membrane fatty acids (Fozo *et al.*, 2004) highlight a few of these adaptations. Their acid resistance and low pH activity has been shown in oral plaque (McLean *et al.*, 2012) and *in vitro* (Tanner *et al.*, 2011) which gives them a competitive advantage and is likely the paramount reason by which they have been reported to dominate a diversity of acidic niches such as caries-associated dental plaque (Kneist *et al.*, 2010). In this study, we identified an *L. fermentum* species (most closely related to the genome sequenced *L. fermentum* IFO-3956) that was highly active at low pH. Changes in gene expression at the lowest pH stage (pH 4.2) were evident as 116 genes were upregulated significantly (*P*<0.05, ⩾1.5-fold cutoff) (Table 2). Down expression of genes were also high at the lowest pH stage (48 genes, *P*<0.05, ⩾1.5-fold cutoff) showing that metabolism shifted significantly in *L. fermentum* as pH changed. As the pH recovered to 5.2, only four to six genes showed a significant change (Table 2) indicating a continued metabolism as the pH recovered.

To maintain intracellular pH at favorable levels during acid stress, *L. fermentum* appears to use an F-Type proton transporting ATPase (subunits K02112, K02113, K02115) as the expression of this ATPase increased twofold at the lower pH stages. Additional indications of adaptation to low pH for *L. fermentum* was that enzymes participating in fatty acid biosynthesis (initiation and elongation pathways, KEGG modules M00082 and M00083, respectively) were upregulated fourfold at the lower pH stages confirming a documented response in increased cell wall biosynthesis to protect the intracellular environment from acid stress (Kajfasz *et al.*, 2011). *L. fermentum* also showed indications of unique adaptations to high lactate accumulations by using energy efficient export of lactate *via* aquaporins instead of using costly active transportation *via* a lactate permease, which most bacteria are limited to (Supplementary Information). Other important functions that were significantly changing across the pH stages for *L. fermentum* are discussed in the Supplementary Information.

### Varied acid stress-related metabolic responses within the *Streptococcus* genus

Members belonging to the *Streptococcus* genus constitute the major population of the oral ecosystem in human and are also predominant in the *in vitro* biofilm model studied here (Edlund *et al.*, 2013). At all pH stages in the *in vitro* biofilm, *S. salivarius* showed gene expression changes most significantly for ammonium and also iron transport proteins that increased 2.8- and 2.9-fold, respectively, as pH dropped (Supplementary Dataset S2,Supplementary Information). In addition to the urease pathway described (Figure 4), the known detoxifying enzyme lactoylglutathione lyase (EC:4.4.1.5) (Korithoski *et al.*, 2007) increased in expression in *S. salivarius* at pH 4.2. This enzyme is involved in the critical detoxification of methylglyoxal, a highly toxic electrophilic glycolytic by-product that reacts with and inactivates intracellular macromolecules, including both proteins and nucleic acids (Inoue and Kimura, 1995). The lactoylglutathione lyase enzyme has also been shown to have important roles in aciduricity and acidogenicity in *S. mutans* (Korithoski *et al.*, 2007). Interestingly, all genes that were upregulated in *S. salivarius* showed the same pattern in *S. vestibularis* suggesting that these species respond in a similar manner to glucose amendment and pH changes. However, *S. vestibularis* showed a higher number of upregulated genes at the lowest pH stage (Table 2) and, therefore, it seems to have a broader metabolic response to acidification than *S. salivarius* (Supplementary Dataset S2). Both species showed considerably high urease activity, glutamate dehydrogenase activity and ammonia gas-transporting activity (discussed previously in the context of alkali-generating pathways, Figure 4). The *Streptococcus* species we studied here showed a highly variable response in several metabolic pathways that were not directly related to alkali-generating processes or acid metabolism (discussed in Supplementary Information).

## Conclusions

Here, we have demonstrated that our *in vitro* model system allows us to study changes of key metabolic processes (that is, carbohydrate utilization, pH stress and pH recovery) in the context of a highly diverse community approaching the diversity of human supragingival plaque. We identified several critical metabolic activities that are possibly responsible for the health-associated pH of recovery, which could also be linked to poorly studied species (for example, *Veillonella* species). Although the urease and ADS pathways are thought to be dominant mechanisms, we found multiple pathways for ameliorating low pH and generating ammonia including some with previously undocumented roles for oral plaque. Gene transcription analyses of individual genomes suggest that the ADS pathway is important in the early stages at neutral pH for some bacteria (for example, *S. mitis* and *L. fermentum*) and that the urease activity is likely the most influential as the pH reaches a minimum. Whereas the activities of glutamate dehydrogenase as well as the serine deaminase may become important for many oral bacteria during the recovery phase given the continued increase in expression of these enzymes and their conserved presence across genera. The unexpectedly high diversity of alkali-generating pathways that we identified here may be a critical factor for an individual's caries susceptibility, which needs to be addressed in future studies. We observed clear discernable signatures in the metabolite profiles and corresponding transcriptional profile for both the states of low pH and also pH recovery. Finally, this study will serve as a solid baseline for defining healthy and disease-like states when sampling the metabolome or transcriptome of highly variable supragingival plaque of human populations.

## Figures and Tables

**Table 1:** Correlation matrix showing high reproducibility between replicate mRNA sequencing libraries for ORF counts

*mRNA library*	*pH 7 Rep. 1*	*pH 7 Rep. 2*	*pH 7 Rep. 3*	*pH 4.2 Rep. 2*	*pH 4.2 Rep. 3*	*pH 5.2 Rep. 1*	*pH 5.2 Rep. 2*	*pH 5.2 Rep. 3*
pH 7 Rep. 1	1.00							
pH 7 Rep. 2	0.99	1.00						
pH 7 Rep. 3	0.99	0.99	1.00					
pH 4.2 Rep. 2	0.80	0.80	0.80	1.00				
pH 4.2 Rep. 3	0.77	0.79	0.78	0.81	1.00			
pH 5.2 Rep. 1	0.80	0.81	0.81	0.91	0.94	1.00		
pH 5.2 Rep. 2	0.80	0.81	0.80	0.84	0.95	0.96	1.00	
pH 5.2 Rep. 3	0.78	0.78	0.78	0.90	0.94	0.99	0.95	1.00

Abbreviation: ORF, open reading frame.

mRNA reads were mapped to the reference-ORF data set consisting of 2 288 459 unique gene annotations. Linear correlation values (*r*-values) are presented on datasets after filtering of ORFs with counts greater than 100 reads.

**Table 2:** Percentage CDS covered by mRNA reads in reference genomes and differential expression at three levels (gene, KO, module) of function

*Species*	*Total*	*%*^a^	*%*^a^	*%*^a^	*Total*	*Gene*		*KO*		*Module*	
	CDS	pH 7	pH 4.2	pH 5.2	%^a^	−4.2/7^b^	5.2/4.2^b^	4.2/7^b^	5.2/4.2^b^	4.2/ 7^b^	5.2/ 4.2^b^
SA	1985	99	99	99	99	228/163	2/0	122/78	1/0	17/15	2/0
SV	1973	96	96	96	96	316/402	0/0	118/142	0/0	19/17	1/0
S.sp	1961	92	92	92	94	154/164	0/0	81/54	1/0	12/10	2/0
ST	2056	90	89	89	91	266/342	0/0	150/189	0/0	18/15	1/0
SP	1950	98	98	98	98	51/20	0/0	11/10	0/0	16/6	0/0
SM	1832	95	96	96	97	47/3	12/2	72/8	0/0	1/0	0/0
SS	1963	94	95	96	98	15/25	0/0	7/6	0/0	0/0	0/0
SAg	2125	69	79	81	84	197/39	0/0	122/25	0/0	20/10	0/0
VA	1882	92	98	99	100	55/16	0/0	41/15	0/0	12/5	0/0
VD	1862	81	86	91	94	47/3	12/2	72/8	0/0	12/2	0/0
LF	2029	88	90	92	94	116/48	6/4	81/37	1/3	16/5	1/1
K.sp	5296	69	63	76	83	55/26	0/0	32/12	0/0	16/6	0/0
GH	1682	79	83	84	90	3/1	0/0	2/0	0/0	0/0	0/0
F.sp	2259	86	80	81	90	2/7	0/0	1/0	1/0	0/0	0/0

Abbreviations: CDS, coding DNA sequences; F.sp, *Fusobacterium* sp.; GH, *Gemella haemolysans*; K.sp, *Klebsiella* sp.; KO, KEGG ortholog; LF, *Lactobacillus fermentum*; SA, *S. salivarius*; SAg, *S. agalactiae*; SM, *S.mitis*; SP, *S. parasanguinis*; SS, *S. sanguinis*; S.sp, *Streptococcus* sp.; ST, *S. thermophilus*; SV, *S. vestibularis*; VA, *Veillonella atypica*; VD, *V. dispar*. Total CDS, number of CDSs in reference genomes.

a Percentage of CDS covered by mRNA reads in individual reference genomes.

b The numbers of upregulated and downregulated genes between pH stages (pH 4.2 vs 7 and pH 5.2 vs 4.2) for each KEGG-annotation level (gene, KO group and module) separated by forward slash symbols. Threshold values for differential expression analyses for upregulated functions: log_2_ fold change ⩾0.6, *P*-value: ⩽0.05; downregulated functions: log_2_ fold change ⩽−0.6, *P*-value: ⩽0.05.
