# Supplementary figures and images for: Meta-omics uncover temporal regulation of pathways across oral microbiome genera during in vitro sugar metabolism
Source: ISME J. 2015 May 29;9(12):2605–19. doi: 10.1038/ismej.2015.72 (PMC4817640; doi:10.1038/ismej.2015.72)

ORF level normalized mRNA read counts

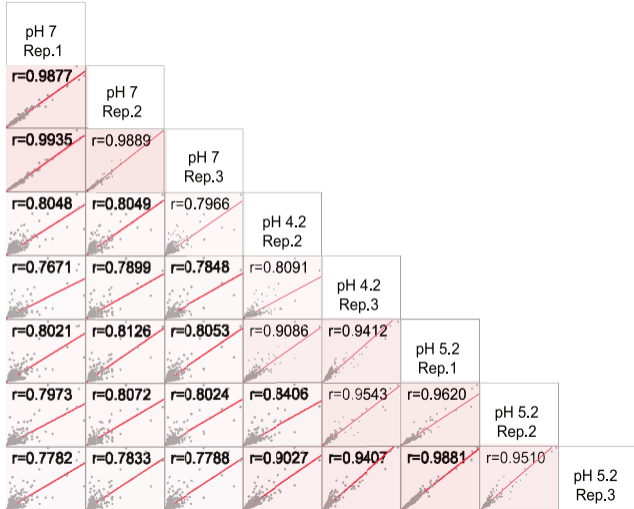

ORF level normalized mRNA read counts

Supplement: Supplementary Figure S1 [file ismej201572x1.pdf]

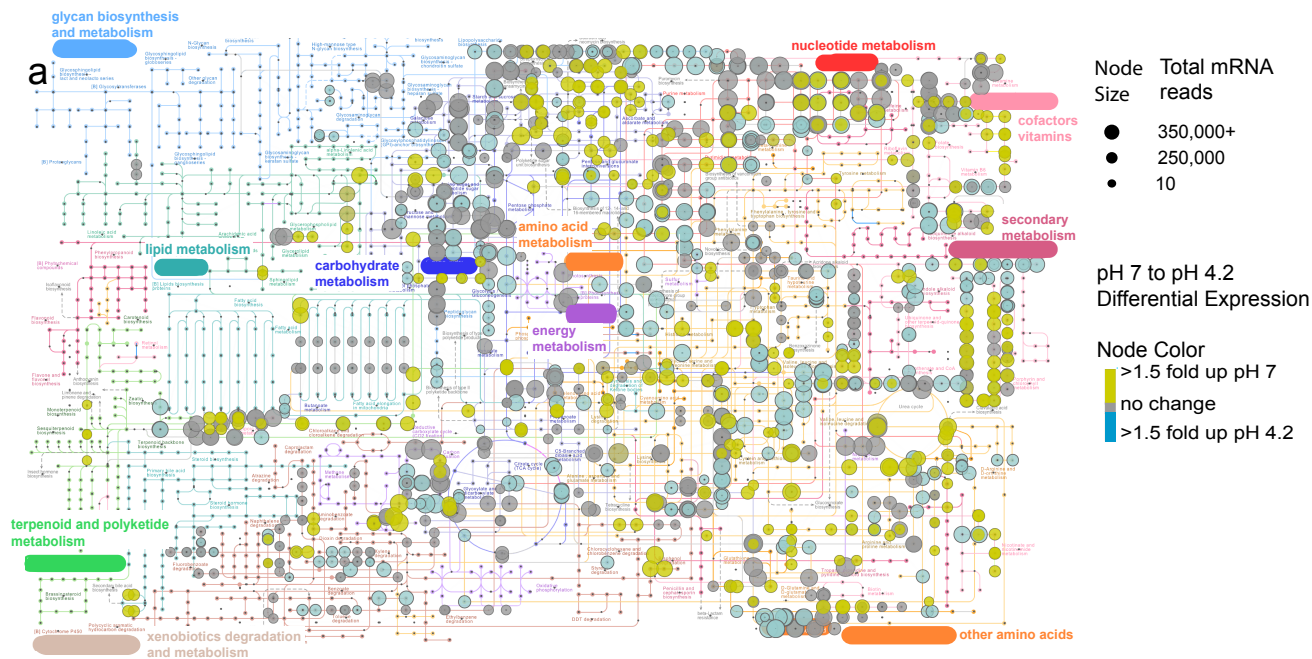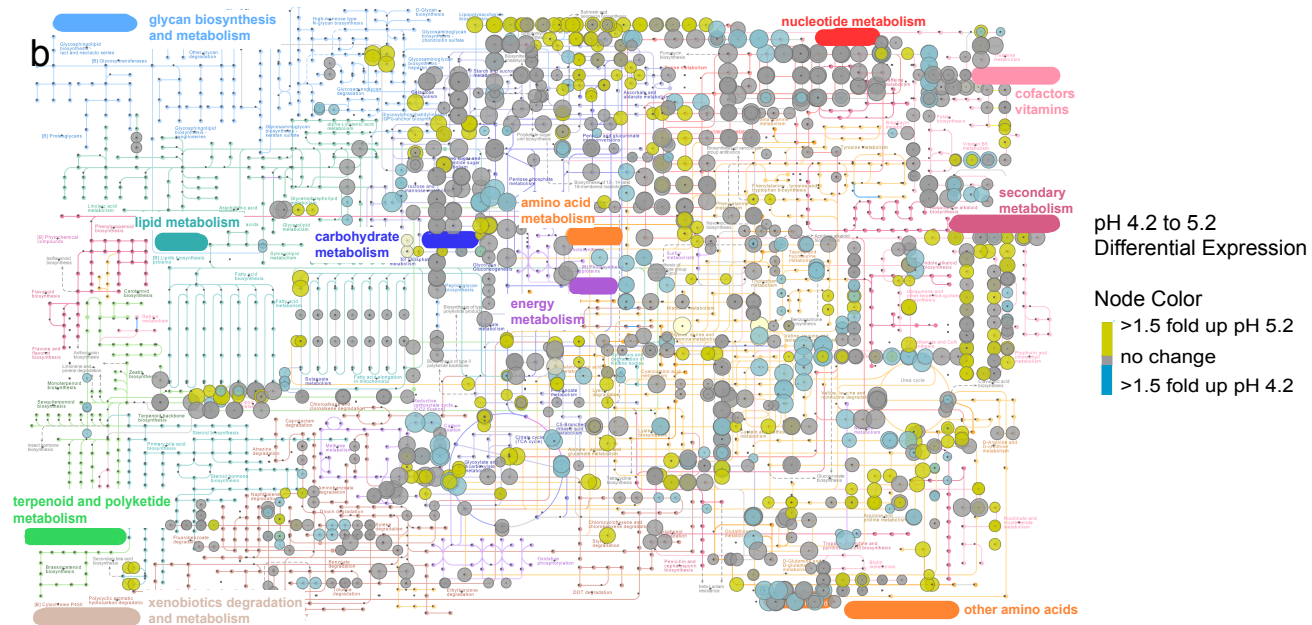

Supplement: Supplementary Figure S2 [file ismej201572x2.pdf]
