# Supplementary material for: Meta-omics uncover temporal regulation of pathways across oral microbiome genera during in vitro sugar metabolism
Source: ISME J. 2015 May 29;9(12):2605–19. doi: 10.1038/ismej.2015.72 (PMC4817640; doi:10.1038/ismej.2015.72)
Supplement: Supplementary Information [file ismej201572x7.doc]

**Supplementary Information**

This supplementary information contains the following files that are referred to throughout the main manuscript:

1) **Supplementary Text** section that is cited several times in the ‘Results and discussion’ in the main manuscript. This text contains additional information on: temporal metabolic activities at a global community level, metabolic activities associated with the key community members (*Veillonella atypica*, *Veillonella dispar*, *Lactobacillus fermentum* and active *Streptococcus* species) as well as detailed gene expression analyses of the ADS-alkalinization pathway.

2) **Supplementary Materials and methods**. Additional details are presented here regarding:

- SHI growth medium and growth-well preparation

- Sample collection, mRNA isolation, mRNA synthesis and sequencing of *in vitro* biofilms

- Analyses of reproducibility of mRNA sequencing libraries at community transcription level

- Generation of a non-redundant read mapping dataset for functional annotation of metatranscriptomic data

- Global transcriptomic profiles and analyses of bacterial growth across pH stages

- Primary metabolite analyses with GC-TOF mass spectrometry

-Global small molecule network analyses with liquid chromatography mass spectrometry

3) **Supplementary Figure S1**. Correlation matrix of DESeq normalized mRNA libraries at all pH stages. Correlations were based on mapping results of replicate mRNA libraries onto the non-redundant ORF dataset. Pearson similarity scores (r) are indicated (1 correspond to identical samples). See main text for description of statistical analysis procedure.

4) **Supplementary Figure S2.** Differential expression of KOs between the different pH stages distributed according to their KEGG pathway. Fold changes (node color) and the total mRNA read counts (node size) are visualized at the KO level for each pH stage by mapping to a network of nodes representing the KEGG-pathways.

5) **Supplementary Table S1**. Primary metabolites from the extra- and intra-cellular biofilm environment. Spreadsheet 1: Replicate samples of annotated and unannotated metabolites analyzed with GC-TOF at different pH stages. Spreadsheet 2: Absolute concentrations (µg/ml) of arginine (Arg) and lactic acid (L.A) as determined by GC-TOF using standard solutions.

6) **Supplementary Table S2**. Quality trimming and mapping results of replicate mRNA libraries from pH 7, 4.2 and 5.2. Library in grey was excluded from all analyses in this study since its mapping success was lower than for other libraries and the variation of mRNA reads indicated it did not have a comparable quality.

7) **Supplementary Dataset S1**. Relative abundance of alkali-generating pathways in reference genomes (first spreadsheet). Differential gene expression values of DESeq-normalized mRNA read counts at KEGG orthology (KO) group level. Average DESeq values for pH stages 4.2, 7 (second spreadsheet) and 5.2 and 4.2 (third spreadsheet) are presented next to their corresponding fold change, log_2_fold change, p-value and adjusted p-value (FDR corrected) and KO-group annotation.

8) **Supplementary Dataset S2**. Differential gene expression values of DESeq normalized counts of mRNA read mapping to reference genomes. Each spreadsheet represents a specific level of KEGG annotation (gene-, KO, or modular level) for the reference genomes when comparing gene expression between pH 4.2 vs 7 and pH 5.2 vs 4.2.

**Supplementary Text**

*Temporal metabolic activities of key community members as inferred from read mapping to reference genomes*

In order to determine the relative activity of a particular species, mRNA library size was normalized for total genome read counts considering all mapped reference genomes and is used as one measure for the change of activity per genome in read abundance over time (Supplementary Dataset S2). The experiment was designed to only provide minimal amounts of nutrients and carbon source to the pre-grown cells in the biofilm community and therefore effectively minimize cell division. Therefore, by using a differential expression analyses approach for individual genomes it was possible to quantify the changes in expression of genes and the metabolic pathway activities across 14 individual genomes as pH varied. Given that the pH reached 4.2, it is reasonable to assume that species such as *Fusobacteria* sp., which showed a 6% overall decrease in reads as the pH dropped (Supplementary Dataset S1), could be inhibited and that their acid tolerance will dictate if they are able to transcribe genes effectively during this stress. Conversely there is a net increase in reads for known acid tolerant species such as *L. fermentum* and *S. parasanguinis*. The overall activity within each representative species is also reflected in the numbers of significantly differentially expressed genes, KO-groups and modules during the pH drop (pH 4.2/pH 7) and subsequent recovery (pH 5.2/pH 4.2) (Table 2).

*Community Level Expression Profiling Results*

The most dramatic fold changes represented the acetyl-CoA synthase (EC: 6.2.1.1) (50 fold increase), a high-affinity nickel transport protein (K07241) (26 fold increase), a maltose/maltose dextrose transport system (K10108) (21 fold increase) (Supplementary Dataset S1). Acetyl-CoA synthase is involved in multiple pathways ranging from propanoate metabolism to biosynthesis of secondary metabolites and its diverse roles can therefore not be predicted exactly in this study. However, the positive response of acetyl-CoA synthase could possibly be attributed to an increase in fatty acid biosynthesis where acetyl-CoA serves as a substrate. This enzymatic reaction was previously observed to increase in bacteria as a response to acid stress (Budin-Verneuil et al 2005). A study show that fatty acids are directed to cell membrane biosynthesis, leading to an increase in cell membrane thickness, which serves as a as a protection from low pH (Budin-Verneuil et al 2005). Furthermore, an L-tartrate dehydratase (K03780) and an Amt ammonium transporter (K03320) were up-regulated (~15 fold) at the lower pH stages. The Amt transporter is important in alkali-generating processes and will be discussed later. This was also the case for a glutamate dehydrogenase enzyme that was significantly upregulated at pH 4.2. Two-component systems (TCS) belonging to the LytT, OmpR NarL and CitB families were up-regulated 4-12 fold at pH 4.2 (Supplementary Dataset S1). TCS are involved in a vast diversity of regulatory responses to external stimuli in bacteria (Gao and Stock 2009) and their overlapping responses to acid stress have been documented previously (Lévesque et al 2007, Suntharalingam et al 2009). Studies show that TCS are important in bacteria sensing acidic conditions as mutations in TCS genes result in acid sensitive phenotypes (Ajdic et al 2002, Li et al 2002). An F-type proton transporting ATPase was up-regulated seven fold showing that the community members employed the well-known proton translocation mechanism to maintain intracellular pH at optimal levels (Bender et al 1986, Bender and Marquis 1987). As pH recovered to 5.2, 121 KO-groups were changing in activity significantly following false discovery rate correction (Materials and methods) showing that gene transcription activities were more similar between the lowest pH stages (4.2) and the recover pH stage (5.2) as compared to the neutral stage and the lowest pH stage (Supplementary Figure S2). However, by including all significant and non-significant KO-groups that changed 1.5 fold (either up- or down-regulated), 651 KO- were up-regulated at pH 5.2 while 498 genes were down-regulated (Supplementary Figure S2b). Focusing on the statistically significant changes (p≤0.05), of up-regulated KO-groups at the pH recovery stage; an agmatinase (K01480), a nitrate reductase (K00370) and a lactate transporter belonging to the LactP family (K0330) were up-regulated (Supplementary Dataset S1). The agmatinase is responsible for the conversion of agmatine to putrescine and urea and could therefore be an important part of the pH recovery. Also, the nitrate reductase produces ammonia via dissimilatory nitrate reduction and may therefore also be important in this process. A KO-group that was significantly downregulated belonged to a carbonic anhydrase (K01674), which indicate that the conversion of carbon dioxide and water to alkaline bicarbonate may be of importance early in the pH-recovery process (Supplementary Dataset S1).

*Metabolic activities of key species*

*Veillonella atypica* and *Veillonella dispar.* Nitrate reduction, which is catalyzed by the nitrate reductase enzyme (EC:1.7.99.4) in the oral cavity is regarded as an efficient host defense against pathogens since it often results in the formation of nitrite and limits the growth of acidogenic bacteria as a result of the production of antimicrobial oxides of nitrogen (Doel et al 2005, Duncan et al 1995). This activity was up-regulated at the recovery stage for both *Veillonella* species (~2 fold change) which is in line with previous observation that *V. dispar* and *V. atypica* make major contribution to host-beneficial nitrate reduction in the oral cavity (Doel et al 2005, Duncan et al 1995). Nitrite and nitrate transporting proteins were constitutively expressed at low levels at neutral pH and at the lowest pH stage for both *Veillonella* species. However at the recovery stage they were up-regulated in *V. dispar* suggesting that this species is transporting nitrite that can be further reduced to nitric oxide by other bacteria in the community (no nitrite reduction related enzymes were expressed in *Veillonella*). Also, both *V. dispar* and *V. atypica* showed upexpression of genes encoding ferrous iron transport proteins-A (K04758) and -B (K04759) (7.9 and 4.6 fold changes, respectively) suggesting that they are effective iron scavengers in the biofilm community at the lowest pH stages which may give them growth advantages over other bacteria that are less adapted to acid-stress. The panhothenate (Vitamin B5) pathway (KEGG module M00119) was significantly up-regulated and increased 10 fold at the lower pH stages for both *Veillonella* species (Supplementary Dataset S2). In *V. atypica* a proton transporting ATPase (K02108) that act on acid anhydrides and transports protons out of cells (KEGG pathway EC: 3.6.3.14) and a hydrogenase (K06281, K06282; small and large subunit, respectively) that generates hydrogen gas (KEGG pathway EC:1.12.99.6) were also significantly up-regulated (2.4 and 2.5 fold, respectively) at the lowest pH stage indicating an important mechanism for maintaining intra-cellular pH levels in an acid stressed environment (Supplementary Dataset S2).

*Lactobacillus fermentum.* In addition to the known lactate utilizers such as *Veilloniella*, some bacteria can switch to lactate utilization in order to continue metabolizing at low pH as glucose becomes limiting or inhibited, which has been observed in anaerobic conditions when *L. plantarum* and *L. pentosus* use citrate as an electron acceptor (Lindgren and Dobrogosz 1990). Other lactate bacteria such as *L. brevis* and *L. buchneri* oxidize lactate by using glycerol as an electron acceptor while producing acetate, 1,3-propanediol and carbon dioxide. In our study intracellular glycerol decreased 3 fold as lactate concentration increased 8 fold at the lowest pH and another 1.6 fold at the recovery pH showing that lactate fermentation and degradation may occur in parallel however, lactate fermentation seem to be more dominant in low pH (Figure 1; see lactate concentrations in Supplementary Table SI). Interestingly, in *L. fermentum,* the lactate dehydrogenase activity was transcribed at high levels at neutral pH (Supplementary Dataset S2) when lactate concentration was relatively low both extra- and intracellularly of the *in vitro* biofilm (Supplementary Table SI) and down-regulated 2-3 fold (but still active) at the lower pH stages when lactate was plenty. Also, *L. fermentum* harbored both D-lactate and L-lactate dehydrogenase enzymes however transcription activity was only present for the D-stereoisomer, which was not the case for any other species we studied here (i.e. other species transcribed L-lactate dehydrogenase only). Transportation of lactate across the membrane is well known to be carried out by the lactate permease and is driven by a proton motive force. However, this mechanism was not expressed in *L. fermentum* at any pH stage. Instead a more energy efficient process that recently was shown to contribute to lactate fluxes across *Lactobacillae* cell membranes via glycerol-facilitating aquaporins (encoded by *GlpF* genes) (Bienert et al 2013) could be identified as up-regulated 101 fold at the lowest pH and then down-regulated 2 fold as pH recovered (Supplementary Dataset S2). The *GlpF* genes are unique to the group of *Lactobacilli* and show that no extra energy has to be spent on removing acidic lactate from the intracellular environment as compared to other bacteria that require a proton motive force to be built up prior to removal of the acid from the cell. Also, a nitrate reductase (subunits K00370, K00371, K00372) was up-regulated 5.6 fold at the lowest pH stages, which confirms that *L. fermentum* is involved in host-beneficial nitrate reduction as well as the *Veillonella* species.

*Streptococcus.* In all *Streptococcus* genomes that were analyzed here, mobile genetic elements were up-regulated significantly as pH decreased to 4.2 and recovered to 5.2 (Supplementary Dataset S2). This suggests that DNA-rearrangements within *Streptococcus* genomes are important, which also were proposed in previous studies where bacteria used DNA rearrangements to increase their genetic diversity and therefore their chances for next generation’s survival in an extreme environment (Arber 2004, McMillan et al 2007, Sørensen et al 2005). Also, all analyzed *Streptococcus* species (*S. salivarius, S. parasanguinis, S, agalactiae, S. thermophilus, S. versitbualris, S.* sp C-150), except *S. mitis,* showed significant upexpression of a gene encoding manganese superoxide dismutase at the lower pH stages suggesting that these species were actively defending themselves against endogenous oxygen species produced by themselves or other bacterial community members (Lisher and Giedroc 2013). In parallel, for the same species, the manganese ABC- transport protein encoded by the *SitA*, *SitB* and *SitD* genes were significantly up-regulated showing that manganese was actively transported into the cells to aid in the protection mechanism against oxidative stress. Moreover, *S. salivarius, S. thermophilus, S. parasangunis. S. sangunis* and *Streptococcus* sp. C-150 showed significant upregualtion of the *MntH* gene, a proton-dependent manganese transporter.

At the pH recovery stage gene transcription increased significantly for genes encoding a phage-associated cell wall hydrolase, an acetoin dehydrogenase, a manganese ABC transporter and a pore forming peptide (a putative bacteriocin) indicating that *S. salivarius* uses acetoin as an energy source in this environment to form NADH and that pathogenic interaction with phage and other bacteria has increased at this stage (Supplementary Dataset S2). A unique feature for the *S. vestibularis* genome was that it showed a drastic response in iron uptake at pH 4.2 and pH 5.2 as an ABC transporter responsible for iron uptake increased ~66 fold in expression when compared to neutral pH (Supplementary dataset S2). Unique features for the *Streptococcus* sp. C-150 genomes was that a pore forming peptide (a putative bacteriocin) was up-regulated at pH 4.2 as well as a pantothenate transport protein that increased in expression 6 fold showing that the import or export of vitamin B5 is critical at this low pH stage for this particular species (Supplementary Dataset S2).

*Highly diverse activities of alkali-generating pathways within oral bacteria*

The arginine deiminase system (ADS) is known to be tightly regulated (Nascimento et al 2014) since it could deplete arginine pools for protein synthesis and high ammonia could cause cell death. The ADS pathway is repressed by glucose (catabolite repression) (Stalon et al 1982) and appears to require low pH and anaerobic conditions for optimal expression in some oral streptococci (Dong et al 2004) and lactobacilli (Budin-Verneuil et al 2005). Our data showed these enzymes were highly active at neutral pH without glucose within certain species and drastically decrease in activity when glucose was added (Figure 4). Regulation by transcriptional and post-transcriptional mechanisms in *S. gordonii* has been shown (Dong et al 2004) however it is also been suggested that it may not be the same for every ADS-positive organism, which is what our data indicate. *S. parasangunis* and *S. mitis*, harbored all genes encoding the ADS according to genome annotations. However, the pathway was regulated differently (Figure 4) as *S. mitis* showed induction of the last step in the ADS pathways where ornithine is converted to ammonia by the carbamate kinase at pH 4.2 while *S. parasanguinis* showed constitutive expression at neutral pH and pH 4.2 (Figure 4). Also, *S. mitis* was the only species that showed up-regulation of the arginine/ornithine antiporter at low pH (Figure 4). All reference genomes, except *Fusbacterium* sp., harbored the ornithine carbamoyl transferase (EC:2.1.3.3) encoding gene in the ADS pathways however the critical arginine deiminase (EC:3.5.3.6) and carbamate kinase (EC:2.7.2.2) were only present *in L. fermentum*, *S. parasangunis* and *S. mitis*. Interestingly, within the *Veillonella* species, carbamate kinase activity increased as pH decreased indicating these two species are actively converting ornithine into ammonium and carbon dioxide at pH 4.2 which is likely important for the pH recovery (Figure 4).

By targeting absolute arginine concentrations at the different pH stages with GC-MS we could verify that arginine was present at pH 7 externally and was low internally. After glucose was added, the extracellular arginine pool decreased (43 fold) as the internal pool increased (24 fold) (Figure 4). Gene transcription data showed that arginine import may occur at pH 7 either via up-regulated arginine/ornithine antiporter (*L. fermentum, S mitis, S. parasanguinis*) or via an arginine permease (*S. salivarius, S. vestibularis, V. atypica*) (Figure 4). The intracellular pool of arginine was low at pH 7 whereas citrulline and ornithine were initially high at pH 7 indicating the pathway was perhaps inactive. Consistent with the initiation of the pathway, ornithine and citrulline were rapidly depleted after the glucose pulse and arginine import. This is also in agreement with the fact that arginine import is stoichiometric with ornithine export (Driessen et al 1989). The arginine concentrations in our *in vitro* biofilm system corresponded to the natural concentration of arginine in saliva (~20µM) and therefore, the results that are presented here are representative for arginine related metabolic processes. It is of note that ADS pathway in *L. fermentum*, including the antiporter was highly up-regulated at pH 7 which partly could explain its tolerance to rapid acidification and the observed high activity at pH 4.2 (Figure 4).

*S. parasangunis* and *S. mitis*, harbored all genes encoding the ADS according to genome annotations. However, the pathway was regulated differently (Figure 5) as *S. mitis* showed induction of the last step in the ADS pathways where ornithine is converted to ammonia by the carbamate kinase at pH 4.2 while *S. parasanguinis* showed constitutive expression at the same levels at neutral pH and pH 4.2 (Figure 5). Also, *S. mitis* was the only species that showed up-regulation of the arginine/ornithine antiporter at low pH (Figure 5). All reference genomes, except *Fusbacterium* sp., harbored the ornithine carbamoyl transferase (EC:2.1.3.3) encoding gene in the ADS pathways however the critical arginine deiminase (EC:3.5.3.6) and carbamate kinase (EC:2.7.2.2) were only present *in L. fermentum*, *S. parasangunis* and *S. mitis*. Also, in the two *Veillonella* species, carbamate kinase activity increased as pH decreased showing that these two species are actively converting ornithine into ammonium and carbon dioxide at pH 4.2 which is likely important for the pH recovery as well (Figure 5).

To address which of the identified alkali-generating pathways could be the major mechanism for pH recovery we combined analyses of the relative abundance of each enzyme from our annotated metagenomic assemblies from the *in vitro* biofilms (Edlund et al. 2013) with the existing copy numbers of each enzyme in the sequenced reference genome by using the comparative pathway tools in PATRIC (Pathosystem Resource Integration Center) (Wattam et al 2014). From this data we first calculated the relative genetic potential in the *in vitro* community, per bacterial genome, that can contribute to pH recovery and generated a table of relative abundance of related enzymes of each pathway (see first spreadsheet in Supplementary Dataset S1). Secondly, we compared this data with our DESeq normalized metatranscriptome data representing the reference genomes (Supplementary Dataset S2). This allowed us to predict which bacterial species and pathways may be major contributors of pH recovery.

**Supplementary Methods**

*SHI growth medium and growth-well preparation*

SHI medium (1 L) (Tian et al 2010) was prepared as follows: 10 g protease peptone (Fisher), 5 g trypticase peptone (BD Bacto), 5 g yeast extract (BD Bacto), 2.5 g KCl, 10 ml hemin solution (from 50 mg hemin stock, Sigma), 0.06 g urea, 0.17 g arginine, 2.5 g mucin (type III, porcine, gastric, Sigma), 990 mL distilled water. This solution was autoclaved in 121°C for 15 min, cool to 50°C: 1 mg vitamin K (Alpha Aesar, dissolved in water and filter sterilized) 50 mL sheep blood (CS1122, Colorado serum company), I mL NAM solution was added after cooling. NAM stock solution is prepared as follows: 100 mg N-Acetyl Muramic Acid (Sigma) was dissolved in 10 mL distilled water. The stock solution was then filter sterilized and stored in -20°C.

Cell-free saliva for coating growth-well surfaces was obtained by centrifugation of the pooled saliva at 2,600 g for 10 min. 200 uL of the cell-free top layer was withdrawn with a sterile pipette and used to coat each bottom surface in a 24-well plate by pipetting saliva up and down until bottom surfaces were completely covered. The plate was dried in 37°C for 30 minutes and moved to a flow hood UV- chamber for 45 minutes to allow cross-linking of saliva proteins.

*Sample collection, mRNA synthesis and sequencing of in vitro biofilms*

Samples for mRNA were collected at designated pH stages after removing 0.5 mL of the spent CDM medium and adding 2 volumes of RNAProtect (QIAGEN Inc. USA). Two growth wells containing biofilms that were maintained in RNAProtect were pooled for a single sample, see mRNA extraction below, to represent one single mRNA library replicate. Biofilm biomass from only one growth well was not sufficient to achieve high quality mRNA for downstream processing. A total of three replicate biofilm samples were prepared for each pH stage (neutral pH, pH 4.2 and pH 5.2) in the same manner. Replicate samples that were collected after 2 hrs of carbon starvation at neutral pH are abbreviated pH 7 Rep. 1, pH 7 Rep. 2 and pH 7 Rep. 3. Samples collected at 6 hrs and 9 hrs after glucose amendment are abbreviated pH 4.2 Rep. 1, pH 4.2 Rep. 2, pH 4.2 Rep. 3 and pH 5.2 Rep. 1, pH 5.2 Rep. 2, pH 5.2 Rep. 3, respectively. The biofilm/RNAProtect-suspensions for each replicate were immediately mixed by vortexing for 5 s and incubated in room temperature for 5 min prior to centrifugation for 10 min at 5000 x g following manufacturer’s recommendations. The pelleted biofilms were stored at -80°C for one week prior to RNA extraction. Each replicate (in total 9) was subjected to RNA extraction as follows: pelleted and frozen biofilms were thawed on ice in 350 ul lysis buffer (mirVana RNA extraction kit, Life Technologies, Carlsbad, CA). Pellet-biofilm samples representing two growth wells from the same sampling time point were pooled to generate sufficient amounts of mRNA for sequencing. For each time point 3 replicate samples were prepared. Each replicate was transferred to a bead beating tube (PowerBiofilm RNAisolation kit, MoBio Laboratories Inc., Carlsbad, CA) and inserted into a Mini-bead beater (Bio Spec Products, Bartlesville, OK). Samples were homogenized twice at speed setting ‘homogenize’ for 30 s. Samples were put on ice between and after beating events. Total RNA extraction and purification was performed by using the mirVana RNA extraction kit (Life Technologies) and the RNA Clean and Concentrator™ kit (Zymo Research, Irvine, CA), respectively. DNA was removed from the samples by adding 1 ul (2U) Turbo™ DNAse (Life Technologies) and incubation in 37°C for 30 min. After DNA removal, 16S rDNA PCR was performed by using the same protocol and primers as described in McLean et al (McLean et al 2012) for 32 cycles of amplification to verify that DNA was removed. To remove rRNA in total RNA extracts the RiboZero™Magnetic Kit (Epicenter, Madison, WI) was employed according to manufacturer’s directions. mRNA was purified by using the Zymo RNA Clean and Concentrator™ kit (Zymo Research). RNA concentration and integrity was monitored before and after rRNA removal by using the Agilent RNA 6000 Nano Kit (Agilent Technologies, Inc. Santa Clara, CA) and the Agilent RNA 6000 Pico Kit (Agilent Technologies), respectively.

mRNA library from rRNA-depleted RNA was generated by using random-primed mRNA synthesis methods according to the ScriptSeq™v2 RNA-Seq Library Preparation Protocol (Epicenter). Prior to second strand mRNA synthesis the di-tagged mRNA was purified by using the Agencourt AMPure XP system (BeckmanCoulter). Index-reads supplied with the ScriptSeq Kit were added to the libraries, which then were PCR amplified for 15 cycles. RNA-Seq libraries were purified and quantified by using the Agencourt AMPure XP system (BeckmanCoulter) and the Agilent DNA 1000 protocol (Agilent Technologies), respectively. Sequencing of mRNA libraries was performed by using a Illumina HiSeq 2000 platform (100 bp paired end reads).

Using each sample's individual barcodes, the Illumina data was deconvolved into the respective samples. After trimming the barcodes, low-quality and short sequences were removed by using the CLC ngs-cell program and Perl scripts using the following settings: -c quality score 20, -f phred quality score 33, -m minimum length of sequence to keep after filtering 55 bp (Supplementary Table S2). A Dust Masker program also was used to mask low complexity parts of the CLC-filtered reads. The Ribopicker program v. 0.4.3 (Schmieder et al 2012) was used to remove 16S rRNA gene fragments (Supplementary Table S2). Settings to identify 16S rRNA gene fragment were: 95% 16S rDNA sequence identity and 96% sequence length.

*Reproducibility of mRNA sequencing libraries at community transcription level*

The generation of high-quality mRNA reads from nine RNA extracts, representing biological replicates from the pH stages; neutral pH, pH 4.2 and 5.2 was successful. Only 5% to 12% of the mRNA reads consisted of rRNA-encoding reads after using the RiboZero rRNA removal method (Supplementary Table S2). After quality trimming and additional rRNA read removal the number high-quality mRNA reads that remained in each library ranged between 20,275,389 to 52,484,463 (Supplementary Table S2).

DESeq normalized counts were calculated both for the ORF dataset based mapping (Supplementary Dataset S1) and the individual genomes based mapping (Supplementary Dataset S2). Comparisons of the numbers of annotations that were either up or down-regulated at the different pH stages was performed to quantify how the activity at both community and genome levels were changing (Table 2). To evaluate reproducibility between replicate mRNA libraries, DESEq-normalized read counts were compared between ORF-level mapping events by linear correlation and r-value calculations (Supplementary Figure S1). Also, by analyzing all replicates of the normalized DESeq data with multivariate statistics (correspondence analyses and multi-dimensional scaling) it was clear that the reproducibility between replicates was very high, except for one replicate representing pH 4.2 (results not shown here). This mRNA library (pH 4.2 Rep. 1) failed our quality control (QC) steps and thus this replicate was not included here. The paired-end mRNA reads from the other two highly similar replicates (pH 4.2 Rep.2 and pH 4.2 Rep. 3) at this pH stage mapped at a high percentage (78% to 82%) to the non-redundant ORF data set (Supplementary Table S2). To evaluate how well the obtained mRNA read libraries represented the reference genomes from a gene coverage perspective, comparisons of numbers of CDS that had mapping mRNA reads to the total number of CDS in each genome were performed for each pH stage (Table 2).

*Generation of a non-redundant ORF dataset for functional annotation*

A total of 3,979,383 high-quality ORFs deriving from 1,521 bacterial genomes were initially selected for this dataset. These ORFs were then combined with *de novo* assembled ORFs from our previously obtained metagenomic sequencing data representing a similar *in vitro* biofilm experiment (Edlund et al 2013) as well as *de novo* assemblies of the cDNA from the metatranscriptomic time point samples. The combined number of ORFs was 5,206,280. To reduce redundancy, ORFs were clustered at 100% identity (over 90% alignment coverage) using cd-hit-est (Li and Godzik 2006), and only cluster representatives were used subsequently (referred to as “non-redundant” ORFs). The clustering resulted in 2,288,459 non-redundant ORFs, and these were annotated by the JCVI prokaryotic metagenomics pipeline (Tanenbaum et al 2010). All trimmed mRNA reads were mapped onto this ORF data set and paired reads restriction was enforced meaning that if paired reads could not be matched to the same ORF, both reads were excluded from the analyses.

*Global transcriptomic profiles*

mRNA reads from each library that mapped onto the annotated ORF data set were assigned additional levels of functions by using the BLAST (Altschul et al 1990) and the best-hit gene assignment (cd-hit 100%) against KEGG. ORFs from the non-redundant data set were classified at the following KEGG levels: Ortholog groups (KO), KEGG module and KEGG pathway. To address if the metatranscriptomic dataset required further normalization for differential bacterial growth during the pH shifts we analyzed the expression within reference genomes, whose activity was either high (i.e. *Streptococcus salivarius*) or changed significantly in their fold change across pH stages (e.g. *Lactobacillus fermentum, Veillonella atypica* and *V. dispar*). Based on the DESeq normalized data we compared all the detected cell division genes (e.g. *FtsE*, *FtsH*, *FtsI, FtsK, FtsQ, FtsW, FtsZ, MraZ*) that were transcribed in the above-mentioned genomes.

*Primary metabolite analyses with GC-TOF mass spectrometry*

Understanding changes of metabolites in a temporal manner between the inside and outside of the biofilm cells required analyses of growth media representing the extra cellular environment [M]_ext_ and the intracellular environment [M]_int_. Growth media and biofilms were therefore collected into separate sampling tubes (Supplementary Materials and methods). 6 replicate samples were collected for each environment at the following pH stages: neutral pH, pH 6.5, pH 4.8, pH 4.2 and pH 5.2. A total of 30 [M]_ext_ replicate samples were submerged in an organic solvent mixture of methanol:acetonitrile:toluene  in a ratio of 50:35:15 prior to freezing and shipping to prevent compound degradation. The abbreviations for the [M]_ext_ and [M]_int_ replicate samples are: pH 7 Rep 1- Rep.7, pH 6.5 Rep 1- Rep.6, pH 4.8 Rep 1- Rep.4.2, pH 4.2 Rep 1- Rep.6, pH 5.2 Rep 1- Rep.6, respectively.

The extra cellular biofilm samples [M]_ext_ were extracted according to protocols at the UC-Davis Metabolmics center without any organic solvents. 50uL of each media sample was dried, derivatized and then injected on the GC-TOF.  Samples from the intracellular biofilm environments [M]_int_ samples were extracted by subjecting biofilms to grinding with liquid nitrogen and additions of extraction solvents (3:3:2 ACN:Isopropanol:H_2_O). The organic material was spun down into a pellet and extraction solvent was removed. The pellets were dried and derivatized by using 3-O-methyloxazepam (MeOX), N-Methyl-N-(trimethylsilyl) trifluoroacetamide (MSTFA) and methyl esters (FAMEs) according to the Metbolomics center’s standard protocol. The relative abundance of primary metabolites from [M]_int_ and [M]_ext_ were analyzed with GC-TOF Mass spectrometry. The profiling data was run through the Fiehn laboratory primary metabolite libraries to identify known and unknown compounds in each replicate. Absolute quantification of glucose, lactate and arginine from [M]_int_ and [M]_ext_ was performed by injecting a known amount each compound into the GC-TOF and then standard curves and absolute concentrations could be calculated. Data were acquired using the chromatographic parameters described in Fiehn et al. 2008 (Fiehn et al 2008). Mass spectrometry parameters were used as follows: a Leco Pegasus IV mass spectrometer was used with unit mass resolution at 17 spectra s^-1^ from 80-500 Da at -70 eV ionization energy and 1800V detector voltage with a 230°C transfer line and a 250°C ion source.

Raw data files from GC-TOF were preprocessed directly after data acquisition and stored as ChromaTOF-specific *.peg files, as generic *.txt result files and additionally as generic ANDI MS *.cdf files. The ChromaTOF vs. 2.32 software was used for data preprocessing without smoothing, 3 s peak width, baseline subtraction just above the noise level, and automatic mass spectral deconvolution and peak detection at signal/noise levels of 5:1 throughout the chromatogram. The BinBase database was employed at the NIH West Coast Metabolomics Center to identify metabolites by using the BinBase algorithm (rtx5) with the following settings: validity of chromatogram (<10 peaks with intensity >10^7 counts ^s-1^), unbiased retention index marker detection (MS similarity>800, validity of intensity range for high m/z marker ions), retention index calculation by 5th order polynomial regression. Spectra were cut to 5% base peak abundance and matched to database entries from most to least abundant spectra using the following matching filters: retention index window ±2,000 units (equivalent to about ±2 s retention time), validation of unique ions and apex masses (unique ion must be included in apexing masses and present at >3% of base peak abundance), mass spectrum similarity must fit criteria dependent on peak purity and signal/noise ratios and a final isomer filter. All thresholds reflected settings for ChromaTOF v. 2.32. Quantification was reported as peak height using the unique ion as default. A quantification report table was produced for both identified and unidentified metabolites.

*Global small molecule network analyses with liquid chromatography mass spectrometry*

The replicate *in vitro* biofilms samples were collected from each pH stage 7, 4.2 and 5.2 and submerged in a 1:1 ratio of methanol:acetonitrile:toluene (50:35:15). Prior to solvent addition the spent CDM growth medium was carefully removed and discarded. Biofilms were resuspended in the solvent mixture and incubated in room temperature for two hours prior to filtering the mixture through chemically inert polytetrafluoroethylene (PTFE) filters (mesh size 0.2 µm) (EMD Millipore, Billerica, MA) and injected for analyses by a nano-mat electro spray ionization robot (Advion, Ithaka, NY) for consecutive spray into the MS inlet of a LTQ 6.4T FT-ICR mass spectrometer (Thermo Finnigan, Thermo Fisher Scientific, Carlsbad, CA). MS and MS^n^ data were acquired in positive ion mode using a Biversa Nanomate 100 (Advion Biosciences, Ithaka, NY) and analyzed using a hybrid 6.4T LTQ-FT mass spectrometer (Thermo Finnigan, Thermo Fisher Scientific, Carlsbad, CA). Data were collected using a data-dependent MS/MS method where an MS scan between 50-1600 *m/*z was followed by MS/MS of the four most intense ions (2 *m/z* isolation width, a normalized collision energy of 35%, and an activation time of 30 ms), which were then added to an exclusion list, allowing for another MS scan followed by MS/MS of the next four most intense ions. Molecular Networking analyses were performed at the UCSD hosted Global Natural Products Social Molecular Networking web server (<http://gnps.ucsd.edu/ProteoSAFe/static/gnps-splash.jsp>). This platform provides an overview of the molecular features in mass spectrometry based metabolomics by comparing fragmentation patterns to identify chemical relationships.  This comparison is based upon the similarity cosine scoring of MS/MS spectra and the visualization of those relationships in a 2-dimensional network in the Cytoscape software. A single chemical species is represented as a node and the relatedness between spectra is represented as an edge. The following settings were applied: ‘ Minimum cosines setting: 0.7; Network TopK: 10; Minimal Matched Peaks: 6, minimum Cluster Size: 1, Run MS Cluster was selected. Network results were visualized with the Cytoscape software v.3.0.2.

**References Supplementary Information**

Ajdic D, McShan WM, McLaughlin RE, Savic G, Chang J, Carson MB *et al* (2002). Genome sequence of Streptococcus mutans UA159, a cariogenic dental pathogen. *Proc Natl Acad Sci U S A* **99:** 14434-14439.

Altschul SF, Gish W, Miller W, Myers EW, Lipman DJ (1990). Basic local alignment search tool. *J Mol Biol* **215:** 403-410.

Arber W (2004). Biological evolution: lessons to be learned from microbial population biology and genetics. *Res Microbiol* **155:** 297-300.

Bender GR, Sutton SV, Marquis RE (1986). Acid tolerance, proton permeabilities, and membrane ATPases of oral streptococci. *Infect Immun* **53:** 331-338.

Bender GR, Marquis RE (1987). Membrane ATPases and acid tolerance of Actinomyces viscosus and Lactobacillus casei. *Appl Environ Microbiol* **53:** 2124-2128.

Bienert GP, Desguin B, Chaumont F, Hols P (2013). Channel-mediated lactic acid transport: a novel function for aquaglyceroporins in bacteria. *Biochem J* **454:** 559-570.

Budin-Verneuil A, Pichereau V, Auffray Y, Ehrlich DS, Maguin E (2005). Proteomic characterization of the acid tolerance response in Lactococcus lactis MG1363. *Proteomics* **5:** 4794-4807

Doel JJ, Benjamin N, Hector MP, Rogers M, Allaker RP (2005). Evaluation of bacterial nitrate reduction in the human oral cavity. *Eur J Oral Sci* **113:** 14-19.

Dong Y, Chen YY, Burne RA (2004). Control of expression of the arginine deiminase operon of Streptococcus gordonii by CcpA and Flp. *J Bacteriol* **186:** 2511-2514.

Driessen AJ, Molenaar D, Konings WN (1989). Kinetic mechanism and specificity of the arginine-ornithine antiporter of Lactococcus lactis. *J Biol Chem* **264:** 10361-10370.

Duncan C, Dougall H, Johnston P, Green S, Brogan R, Leifert C *et al* (1995). Chemical generation of nitric oxide in the mouth from the enterosalivary circulation of dietary nitrate. *Nat Med* **1:** 546-551.

Edlund A, Yang Y, Hall AP, Lihong G, Lux R, He X *et al* (2013). An *in vitro* biofilm model system maintaining a highly reproducible species and metabolic diversityapproaching that of the human oral mcrobiome. *Microbiome* **1**.

Fiehn O, Wohlgemuth G, Scholz M, Kind T, Lee dY, Lu Y *et al* (2008). Quality control for plant metabolomics: reporting MSI-compliant studies. *Plant J* **53:** 691-704.

Gao R, Stock AM (2009). Biological insights from structures of two-component proteins. *Annu Rev Microbiol* **63:** 133-154.

Lévesque CM, Mair RW, Perry JA, Lau PC, Li YH, Cvitkovitch DG (2007). Systemic inactivation and phenotypic characterization of two-component systems in expression of Streptococcus mutans virulence properties. *Lett Appl Microbiol* **45:** 398-404.

Li W, Godzik A (2006). Cd-hit: a fast program for clustering and comparing large sets of protein or nucleotide sequences. *Bioinformatics* **22:** 1658-1659.

Li YH, Lau PC, Tang N, Svensäter G, Ellen RP, Cvitkovitch DG (2002). Novel two-component regulatory system involved in biofilm formation and acid resistance in Streptococcus mutans. *J Bacteriol* **184:** 6333-6342.

Lindgren SE, Dobrogosz WJ (1990). Antagonistic activities of lactic acid bacteria in food and feed fermentations. *FEMS Microbiol Rev* **7:** 149-163.

Lisher JP, Giedroc DP (2013). Manganese acquisition and homeostasis at the host-pathogen interface. *Front Cell Infect Microbiol* **3:** 91.

McLean JS, Fansler SJ, Majors PD, McAteer K, Allen LZ, Shirtliff ME *et al* (2012). Identifying Low pH Active and Lactate-Utilizing Taxa within Oral Microbiome Communities from Healthy Children Using Stable Isotope Probing Techniques. *PLoS One* **7:** e32219.

McMillan DJ, Sriprakash KS, Chhatwal GS (2007). Genetic variation in group A streptococci. *Int J Med Microbiol* **297:** 525-532.

Nascimento MM, Browngardt C, Xiaohui X, Klepac-Ceraj V, Paster BJ, Burne RA (2014). The effect of arginine on oral biofilm communities. *Mol Oral Microbiol* **29:** 45-54.

Schmieder R, Lim YW, Edwards R (2012). Identification and removal of ribosomal RNA sequences from metatranscriptomes. *Bioinformatics* **28:** 433-435.

Sørensen SJ, Bailey M, Hansen LH, Kroer N, Wuertz S (2005). Studying plasmid horizontal transfer in situ: a critical review. *Nat Rev Microbiol* **3:** 700-710.

Stalon V, Simon JP, Mercenier A (1982). Enzymes of arginine utilization and their formation in Aeromonas formicans NCIB 9232. *Arch Microbiol* **133:** 295-299.

Suntharalingam P, Senadheera MD, Mair RW, Lévesque CM, Cvitkovitch DG (2009). The LiaFSR system regulates the cell envelope stress response in Streptococcus mutans. *J Bacteriol* **191:** 2973-2984.

Tanenbaum DM, Goll J, Murphy S, Kumar P, Zafar N, Thiagarajan M *et al* (2010). The JCVI standard operating procedure for annotating prokaryotic metagenomic shotgun sequencing data. *Stand Genomic Sci* **2:** 229-237.

Tian Y, He X, Torralba M, Yooseph S, Nelson KE, Lux R *et al* (2010). Using DGGE profiling to develop a novel culture medium suitable for oral microbial communities. *Mol Oral Microbiol* **25:** 357-367.

Wattam AR, Gabbard JL, Shukla M, Sobral BW (2014). Comparative genomic analysis at the PATRIC, a bioinformatic resource center. *Methods Mol Biol* **1197:** 287-308.
